# A hybrid feature hashing approach with temporal validation for efficient forgery detection of frame duplication

**DOI:** 10.1038/s41598-026-69134-w

**Published:** 2026-09-09

**Authors:** Mona M. Ali, Neveen I. Ghali, Hanaa M. Hamza, Khalid M. Hosny

**Affiliations:** 1 Department of Information Technology, Faculty of Computers and Information, Zagazig University, Zagazig, 44519 Egypt; 2 Department of Information Technology, Faculty of Computers and Artificial Intelligence, Al-Azhar University, Cairo, Egypt

**Keywords:** Forgery video, Interframe forgery, Duplicated frames, ResNet50, Deep learning, MD5 hashing, Engineering, Mathematics and computing

## Abstract

This study presented a hybrid framework for detecting video duplication forgery by leveraging deep learning, hashing techniques, and motion analysis. Two novel approaches were explored to evaluate the trade-off between processing speed and detection accuracy. The first approach employed a window-based strategy, using ResNet-50 for feature extraction and MD5 hashing to identify potential duplicates, yielding fast execution times. The second approach improved upon this by implementing a two-stage validation process: hash-based candidate selection, followed by temporal and structural validation using a gap threshold and optical flow analysis, thereby significantly enhancing detection accuracy. Experimental evaluations on the SULFA, TDTVD, and Fadl datasets using the proposed group-based approach demonstrate key quantitative outcomes, achieving an accuracy of 100%, a precision of 99.91%, and an F1-score of 100% on average using MD5 hashing, surpassing QPCET hashing’s 98.2% accuracy, a precision of 94.6%, and an F1-score of % 90.8%. This ensures reliable detection of duplicate frames and yields a 65.85% reduction in execution time relative to frame-wise processing while maintaining detection accuracy. This paper’s contributions include a robust, scalable video pipeline that achieves state-of-the-art performance on benchmark datasets, a 25-frame-window recommendation that balances accuracy and speed, and validation of MD5’s effectiveness relative to QPCET for forgery detection. These findings advance automated video authentication, with implications for digital forensics, surveillance, and the verification of social media content.

## Introduction

The world around us is not always truthful, as individuals sometimes harbor ulterior motives and seek to distort the truth. In such cases, people often struggle to find a resolution due to lingering doubts. Manipulating videos to convey a false narrative can lead to grave consequences, such as unjustly incriminating innocent individuals or failing to hold genuine culprits accountable. Thus, it is crucial to prioritize developing techniques for detecting forged videos. Video forging, which involves altering video content by copying frames or using editing software to create false scenes, is often employed to falsify or distort information^1^.

**Fig. 1:**
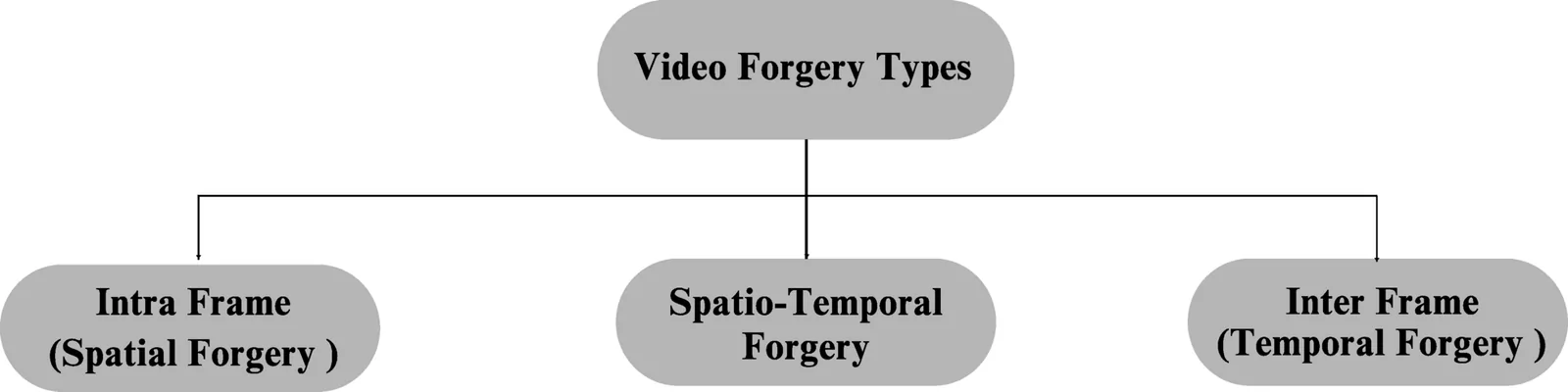
The classification of video forgery.

Video forgery is a complex and multifaceted phenomenon in digital media manipulation. It can be categorized into three primary types: intra-frame, spatio-temporal, and inter-frame forgery, as illustrated in Fig. 1. Intra-frame forgery involves altering individual frames within a video sequence. Techniques in this category may include removing specific objects from a scene, swapping facial features between individuals, or replacing the background with entirely new imagery to construct a misleading visual narrative^1^.

Spatio-temporal forgery involves manipulating both spatial elements and the temporal flow of a video. This may include adjusting frame rates, interpolating intermediate frames to create smooth transitions, or morphing between distinct frames to produce a seamless effect that can mislead viewers^2^.

Inter-frame forgery explicitly addresses the relationships and sequences between consecutive frames. Techniques in this category may include inserting additional frames to alter the video’s pacing, duplicating frames to emphasize specific moments, deleting frames to create narrative jumps, or rearranging frames to disrupt the intended chronological order of the visual content.

Detecting these various forms of forgery presents a significant challenge due to their intricate nature. However, multiple methodologies, including feature-based approaches, machine learning algorithms, and advanced deep learning techniques, are actively employed to identify manipulated videos. These efforts play a crucial role in combating the widespread challenges of misinformation and disinformation in today’s media landscape^3^.

**Fig. 2:**
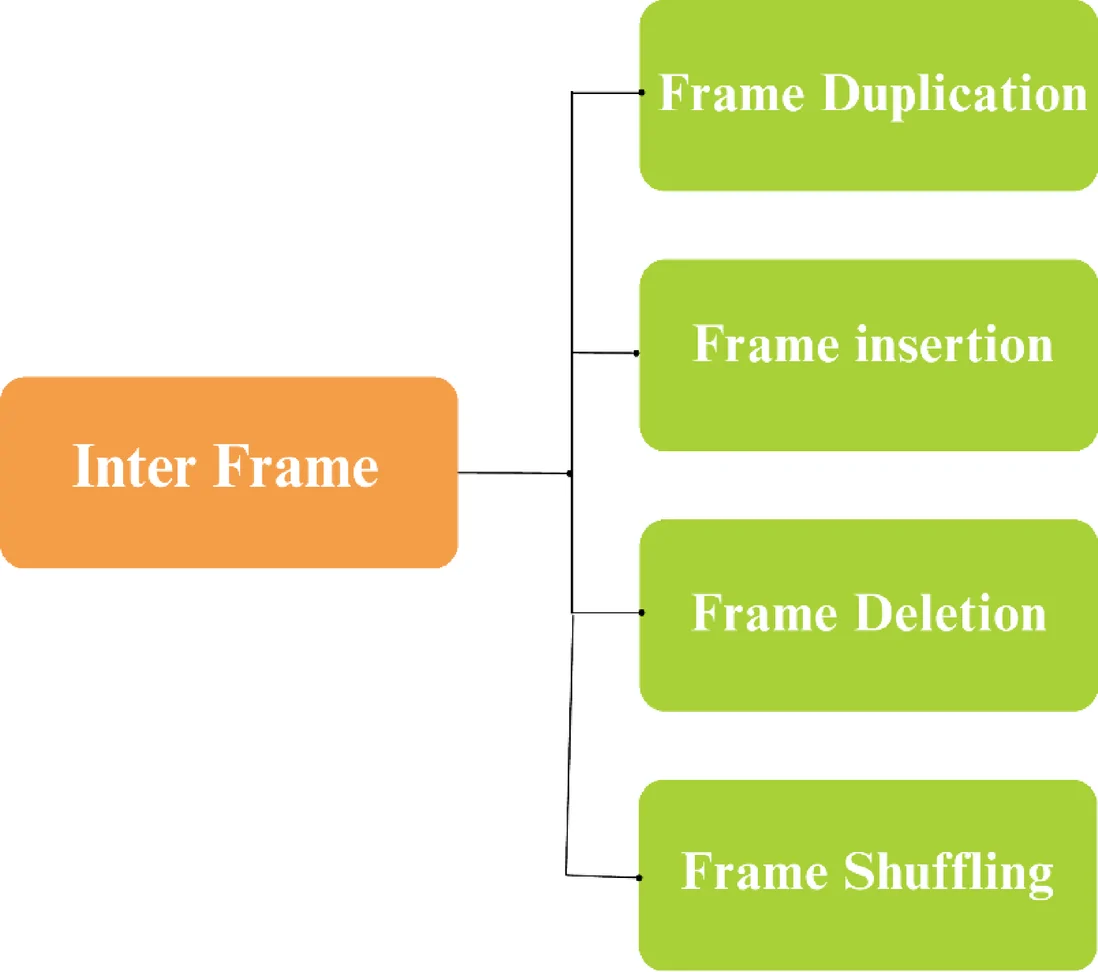
Types of inter-frame forgery video.

Various types of inter-frame manipulation in video forgery include frame duplication, insertion, deletion, and shuffling, as shown in Fig. 2. Frame duplication involves copying and reinserting frames, which creates repetition and distorts the original sequence^4^. Frame insertion adds new frames to introduce or modify content, while frame deletion removes existing frames, potentially concealing events or altering timing. Frame shuffling rearranges the order of frames, changing the chronological flow of actions^5^. In this research, the primary focus is on detecting frame duplication, which is critical for identifying instances of video forgery and ensuring the integrity of visual data.

The research problem of detecting frame duplication in video forgery is that existing methods fail to effectively balance accuracy and computational efficiency. Current techniques often exhibit high false-positive rates, scalability limitations, and inadequate real-time processing, compromising their reliability in critical applications such as legal evidence validation, surveillance systems, and misinformation mitigation. These gaps underscore the urgent need for an effective solution that can robustly handle complex video tampering while maintaining high precision and processing speed^3^.

The proposed method aims to detect duplicated frames in forged videos by using ResNet50 for feature extraction and MD5 hashing to generate unique hash values for each frame. This method extracts high-dimensional features from video frames, creates unique hashes, and compares them to identify original and duplicated frames. Experiments conducted on TDTVD^6^ and other datasets demonstrated high accuracy and efficiency in detecting frame-duplication forgeries using windows. This approach employs optimized processing methods, including deep learning models and hashing algorithms, to significantly reduce execution time while maintaining accuracy and reliability.

The objective of this study is to develop an efficient and accurate framework for detecting duplicated frames in forged videos by combining deep feature representation, feature hashing, and window-based processing. Experiments conducted on the TDTVD dataset and additional benchmark datasets demonstrate that the proposed framework achieves high detection accuracy, recall, and computational efficiency. The main contributions of this work are as follows:

- A novel hybrid frame duplication detection framework is proposed that integrates ResNet50 deep feature extraction with MD5 feature hashing to generate compact and discriminative representations for efficient frame comparison.
- Unlike existing hybrid methods that combine deep learning with motion analysis or optical flow, the proposed approach performs duplication detection using deep feature hashing, significantly reducing computational overhead while maintaining high detection accuracy.
- A window-based matching strategy is introduced to limit frame comparisons within local temporal regions, reducing execution time and improving scalability for long video sequences.
- The proposed framework is extensively evaluated on the TDTVD dataset^6^ and additional benchmark datasets, demonstrating high accuracy and recall, and strong generalization capability across different frame-duplication forgery scenarios.
- Experimental results show that the proposed framework provides an effective balance between detection performance and computational efficiency, making it suitable for practical video forgery detection applications.

This research paper is organized as follows. In the first section, the paper introduces the concept of duplication in interframe forgery videos. In the next section, prior and relevant work are discussed. Section “Experiments and results” outlines the research methodology. The study employed inter-frame forgery videos to evaluate the efficiency of the proposed deep learning-based model. The last section concludes the study.

## Related work

This section reviews the recent advances in video forgery detection, covering conventional handcrafted feature-based approaches, deep learning models, hashing techniques, deep hashing, and self-supervised learning methods. The underlying principles, advantages, and limitations of these approaches are analyzed to identify existing research gaps. This analysis motivates the proposed hybrid framework, which aims to strike an effective balance among computational efficiency, detection accuracy, and robustness in detecting frame duplication forgery.

Patel et al.^7^ introduced a method for detecting inter-frame forgery in surveillance videos by extracting features and using a Convolutional Neural Network (CNN) to classify them. This CNN is highly sophisticated. It not only detects forgery but also pinpoints the exact areas that have been tampered with. Oraibi and Radhi^9^ use a 3D-CNN model to extract spatial and temporal features from video sequences, thereby enhancing forgery detection. It incorporates different frames to capture changes between successive frames, providing crucial insights into potential tampering. The Framework’s performance is evaluated using the UCF101 dataset^8^, which comprises 101 action categories, and a custom dataset of surveillance camera recordings. Munawar et al.^9^ propose a novel deep learning framework that integrates an Inflated 3D (I3D) network with a Siamese-based Recurrent Neural Network (RNN). The I3D network examines both authentic and forged videos to find duplicate frames. The Siamese-based RNN processes video sequences at the sequence level to identify forgery at a much higher level than within-frame boundaries.

Kumar et al.^10^ Introduce a method for identifying multiple video forgeries using CNNs, focusing on analyzing deep features extracted from sequential video frames. The technique uses deep feature correlation coefficients to detect inter-frame forgery rather than directly analyzing the video frames. The model’s performance was tested on the VIFFD^11^ and TDTVD datasets^6^.

Fadl et al.^12^ propose a novel method using Motion Energy Image (MEI) and Histogram of Oriented Gradients (HOG) features. Correlation coefficients are examined using HOG features for identification, while MEI is applied to edge-based images to detect frame duplications and shuffling. We tested the method on several interframe datasets. It was more accurate and efficient than other methods in terms of precision, recall, and processing speed. Future developments aim to improve performance in silent scenes and minimize false positives in scenarios involving rapid motion.

Loukhaoukha^13^ present a novel algorithm for detecting realistic video forgeries that uses QR decomposition for feature extraction and the Minkowski distance for comparison. The method was evaluated on the standard dataset TDTVD^6^ and a self-generated dataset. Akhtar et al.^14^ present an effective framework for identifying and locating tampering in surveillance footage. The first stage utilizes Motion Residual-Based Local Binary Patterns (MR–LBP) and a Support Vector Machine (SVM) to detect tampering, while the second stage employs Optical Flow aggregation and the standard deviation of MR–LBP features to identify tampered areas. It has been tested on the COMSATS Structured Video Tampering Evaluation Dataset (CSVTED) and the TDTVD^6^.

Bakas et al.^15^ propose a two-step forensic technique to detect and localize inter-frame video forgeries, specifically frame insertion, deletion, and duplication. The method utilizes Haralick features to measure the correlation between adjacent frames. In the first step, outlier frames are detected using abrupt changes in the correlation distribution. The second step involves a finer-grained approach to eliminate false positives, thereby optimizing forgery-detection accuracy. Experimental results demonstrate that the proposed method outperforms state-of-the-art techniques, proving its efficiency in detecting and localizing video forgeries.

Singh et al.^16^ utilizes a passive blind video forgery detection approach based on Discrete Cosine Transform (DCT), correlation coefficients, and coefficient of variation. It comprises two algorithms: one for detecting frame duplication based on temporal consistency, and another for identifying region duplication via spatial error localization and statistical variation.

Zhao et al.^17^ introduce a combination of HSV color histogram comparison and SURF feature extraction with FLANN matching for detecting inter-frame forgeries in video. It begins by converting video frames to the HSV color space and computing H-S and S-V histograms for each frame. The method assesses the similarity between adjacent frame histograms using correlation and Chi-square metrics to identify potentially tampered frames. To verify these findings, SURF extracts key points, and FLANN matches these points across frames to confirm the type of forgery. This approach effectively detects frame insertions, deletions, and duplications by analyzing histogram similarity patterns and matching feature points in the altered areas.

MD5 (Message Digest 5) is a cryptographic hash function. Both MD5 and MD4 are components of a series of message-digest algorithms, with subsequent versions designed to supplant their earlier counterparts. The algorithm produces a 32-digit hexadecimal number as its output, which is equivalent to a 128-bit hash value. The methodology primarily relies on 32-bit integers and includes addition, bitwise operations, and rotations. Data validation, authentication, and integrity verification are common uses of MD5 across a wide range of applications^18^. It is frequently employed to hash passwords within online platforms. Additionally, it is used in image forgery detection by generating distinct digital fingerprints for images, thereby facilitating prompt, efficient identification of modifications through fingerprint comparison. When integrated with OpenCV, which analyzes image features, MD5 helps verify image integrity, thereby proving effective at detecting various categories of image forgery.^19^.

For high-volume video forensics, processing full feature maps for every frame is computationally expensive. Recent research uses self-supervised learning to compress frames into semantic hash codes, bridging the gap between deep feature extraction and fast retrieval. Lian et al.^20^ proposed a GAN-based adversarial sampling strategy to find the most challenging, information-dense frames in a video. It then applies hash-based contrastive learning to map these frames into a Hamming space. This allows a forensic system to perform rapid, large-scale searches for duplicated frames by comparing the learned hash codes rather than raw feature maps.

An enhancement to image authentication while preserving color fidelity was proposed in^21^ using a robust color image hashing technique based on the quaternion polar complex exponential transform (QPCET). It employs QPCET moments in feature extraction to mitigate the effects of noise and minor texture variations. After applying Gaussian filtering, images are represented as quaternions. A hash is created and encrypted with a secret key to ensure security. QPCET-based reconstruction enables hash decryption using the secret key, and authentication is performed using Euclidean distance between the decrypted and novel hashes. The experiments demonstrate strong resilience to various forms of attack while maintaining high accuracy compared to previous methods. Different detection methods have been proposed to identify these types of forgeries, such as using the improved Levenshtein distance, which has been employed to enhance the accuracy and efficiency of detecting frame duplication forgeries ^5^

Video forgery detection reveals inherent shortcomings across existing methodologies. Deep neural networks (DNNs), while achieving high accuracy, remain constrained by distributional sensitivity, high computational demands, and limited interpretability. Cryptographic hashing techniques such as MD5 guarantee bit-level data integrity but cannot identify semantic-level manipulations and remain susceptible to collision-based vulnerabilities. Likewise, motion-based detection approaches exhibit instability in static or low-motion scenes and lack scalability for large-scale deployment. These approaches have not effectively balanced generalization, efficiency, and accuracy.

While Transformer-based models have demonstrated significant improvements in detecting video forgeries, challenges remain regarding computational complexity and the need for large training datasets. The generalization of these models across different types of forgeries and video qualities is an ongoing area of research^21^. Traditional methods, although less accurate, offer insights into simpler, potentially more computationally efficient solutions^13^.

To overcome these limitations, this research introduces a hierarchical hybrid framework that integrates different techniques within a unified detection pipeline. The proposed framework employs cryptographic hashing for efficient preliminary screening, deep feature extraction with ResNet50 for high-level semantic representation, and temporal-motion validation via optical flow analysis and cosine similarity for enhanced temporal consistency verification. Duplication forgery detection remains a complex challenge due to the computational demands and the need for robust algorithms that can handle diverse video scenarios.

## Methodology

This section outlines the two experimental approaches developed for detecting video duplication forgery within the proposed hybrid framework, which combines deep learning-based feature extraction with hash comparison and motion analysis. The first approach prioritizes computational efficiency through window-based processing, while the second improves detection performance by incorporating frame-level feature extraction and temporal validation. Both approaches share a common backbone that includes a preprocessing stage to ensure compatibility with feature extraction and integrates deep learning-based feature extraction using ResNet50, MD5 hashing, and structured forgery identification workflows.

### First approach: window-based processing with hash matching

#### Preprocessing

The approach requires a preprocessing stage to ensure that input data is standardized and optimized, thereby enabling accurate feature extraction. By resizing frames to a consistent dimension and converting color formats to align with the model’s requirements, preprocessing enhances the data’s compatibility with ResNet50. Normalizing pixel values helps improve the model’s performance by facilitating faster convergence during feature extraction. Effective preprocessing ensures that variations in raw input data do not undermine the model’s ability to detect meaningful patterns, thereby enhancing the robustness and accuracy of the forgery-detection process.

**Fig. 3:**
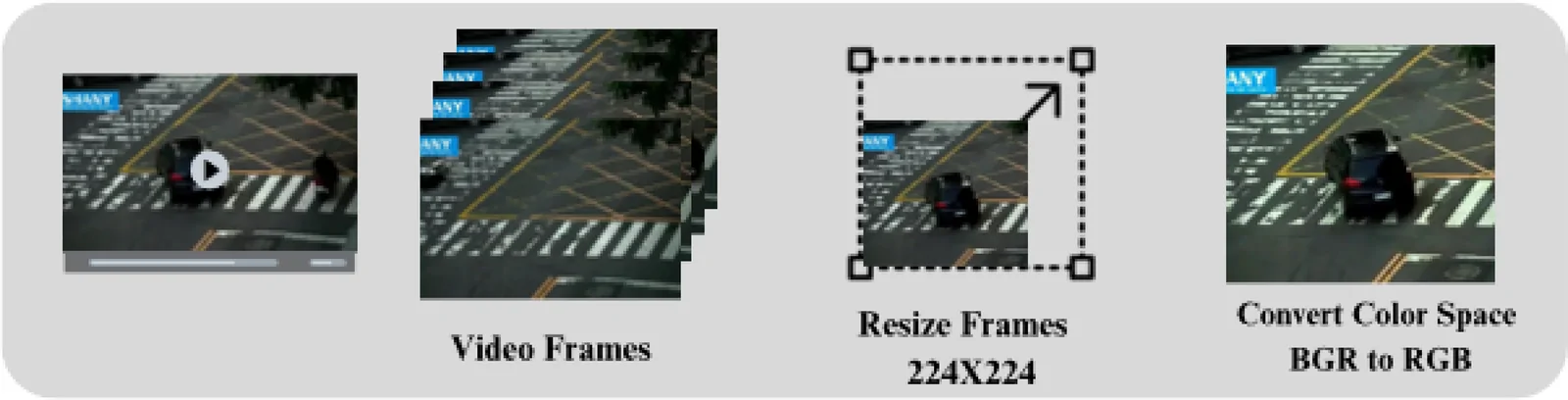
An illustration of the preprocessing stage in the proposed framework.

Each video is processed frame by frame, as illustrated in Fig. 3, with all frames resized to a uniform resolution of 224 × 224 pixels to align with the ResNet50 architecture’s input requirements. The color space is converted from BGR to RGB to match the model’s expected format. To enhance robustness to illumination variations and ensure consistent feature representation, pixel values are normalized using the ImageNet dataset’s statistical parameters: mean [0.485, 0.456, 0.406] and standard deviation [0.229, 0.224, 0.225].

For a video *V* with *N* frames, each frame ${V}_{i}$∈*V* is preprocessed as in Eq. (1):

1 $$ P_{i} = {\mathrm{Preprocess}}\left( {V_{i} } \right) = \left\{ {\begin{array}{*{20}c} {{\mathrm{Resize}}\left( {V_{i} ,224x224} \right) } \\ { {\mathrm{ColorConvert}}(V_{i} , {\mathrm{BGR}} \to {\mathrm{RGB}}) \forall i = 1,2, \ldots ,N} \\ {{\mathrm{Normalize}}(P_{i} ) } \\ \end{array} } \right. $$

#### Feature extraction

**Fig. 4:**
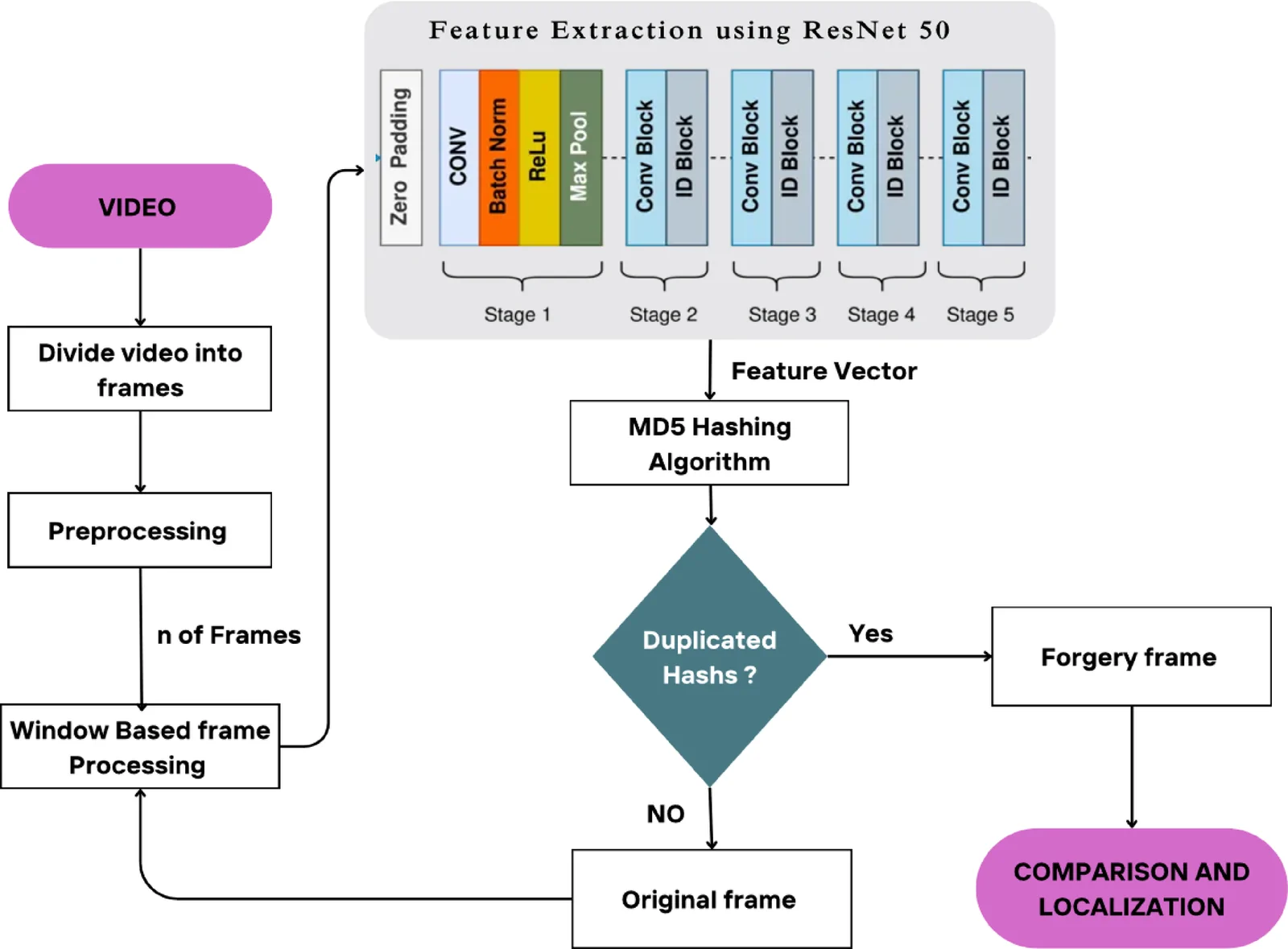
The flowchart of the proposed approach 1 for detecting video duplication forgeries.

Extracting features from video frames is the initial step in the approach. ResNet50 is a CNN architecture known for its depth and efficiency in image classification tasks^22^. It uses residual blocks, which include skip connections that allow information to flow directly from earlier to later layers, bypassing intermediate layers. This helps in training very deep networks without the vanishing gradient problem. ResNet50 consists of multiple convolutional layers, batch normalization layers, ReLU activation functions, and pooling layers for feature extraction, as shown in Fig. 4**.** The extracted features are then processed through fully connected layers to produce the final classification output^23^. This architecture has achieved state-of-the-art performance on various image classification benchmarks, making it a popular choice for computer vision tasks^24^.

The ResNet50 architecture was chosen because it can extract robust and high-dimensional features that describe visual content. Feature extraction using the ResNet50 model is an integral step in obtaining a strong, descriptive representation of individual video frames. ResNet50 is pre-trained on the ImageNet dataset with Stochastic Gradient Descent (SGD) optimizer, a learning rate of *0.1*, a momentum of *0.9*, a weight decay of *0.0001*, and a batch size of 265^25^.The process begins with feeding each preprocessed frame into the model, ensuring that input dimensions match ResNet50’s requirements. The network processes the input through multiple convolutional and pooling layers; each is designed to learn and encode increasingly abstract features.

Key to this approach is the exclusion of the model’s top layers, specifically the fully connected layers typically used for classification tasks. By omitting these, the focus remains on extracting purely feature-based representations rather than predetermined class predictions. The retained layers, up to the global average pooling layer, transform each frame into a high-dimensional vector that captures the complex spatial hierarchies and contextual relationships inherent in the image data.

The global average pooling operation reduces the dimensionality of the feature map while preserving critical spatial information. This layer effectively computes the average of each feature map, converting spatially distributed feature activations into a single 2048-dimensional feature vector. This feature vector encapsulates key attributes of the frame, such as textures, shapes, and patterns, and serves as a unique representation for subsequent processing stages.

The high-dimensional feature vector *Fi* for each frame *i is as* in eq. (2):

2 $$ {\mathrm{Fi}} = {\mathrm{GAP}}\left( {{\mathrm{ResNet5}}0\left( {{\mathrm{Pi}}} \right)} \right) $$

where Pi denotes the preprocessed frame, and GAP denotes global average pooling.

#### Hash generation using MD5 algorithm

MD5 was adopted because the proposed framework requires a fast deterministic hashing function for feature indexing rather than a cryptographically secure hash. The likelihood of an MD5 collision affecting the final detection result is extremely low, and any false candidate generated during hashing is expected to be rejected during subsequent verification. To ensure reliable and efficient comparison of video frames, each extracted feature vector is uniquely encoded using a deterministic MD5 hash. Feature vectors are flattened into one-dimensional arrays and converted into byte streams, which are then subjected to the MD5 hashing process in Eq. (3):

3 $$ Hash \, \left( {F_{i} } \right) = MD5\left( {ByteArray\left( {Flatten\left( {{\mathbf{V}}_{{\mathbf{i}}} } \right)} \right)} \right)\; \in \left\{ {0,1} \right\}^{128} $$

where **V**_**i**_ is the feature vector for frame F_i_.

This cryptographic function iteratively applies a series of bitwise operations, left rotations, and nonlinear functions to compress the input data into a compact 128-bit digest. Padding is used to align the input length with MD5 requirements, allowing processing in 512-bit blocks across four computational rounds. The deterministic nature of MD5 ensures that identical feature vectors yield identical hash outputs, making it an effective mechanism for detecting frame-level duplication by providing a consistent, concise digital signature for each frame.

#### Window-based frame processing

**Fig. 5:**
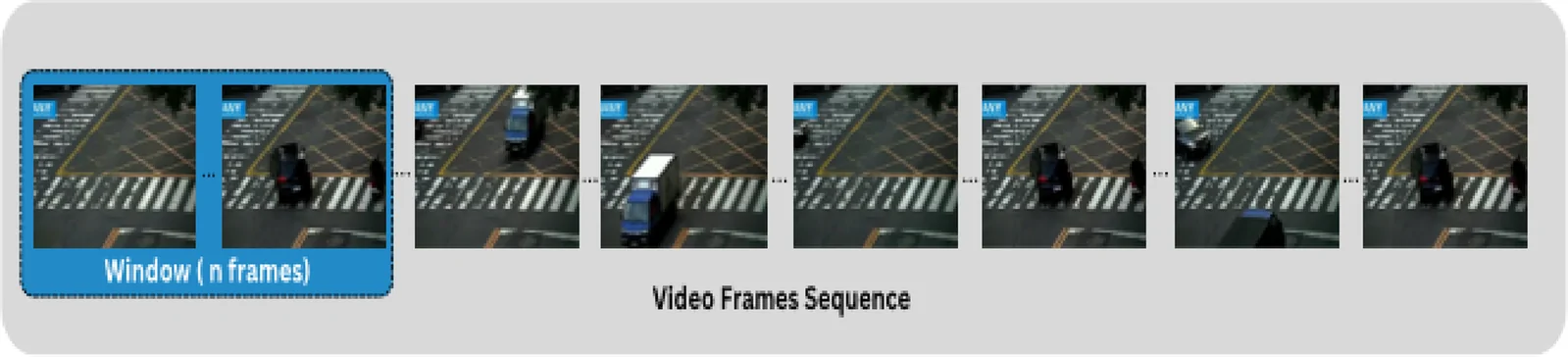
Window-based frame processing.

To enhance computational efficiency and facilitate batch processing, video frames are grouped into windows of size n (10 frames per window), as shown in Fig. 5. Within each window, features are extracted from all frames, and their respective hash values are computed. This window-based approach ensures that frames are processed in manageable batches, optimizing computational resources and reducing processing times for large datasets. By segmenting frames into fixed-size windows, consistent processing is maintained, enabling structured comparisons across different sets of frames and ensuring that every frame undergoes a thorough feature analysis. Windows with fewer frames than the specified size are excluded from processing to prevent inconsistencies and maintain uniformity in feature extraction, as illustrated in Fig. 4.

Duplicate detection and comparison are performed by analyzing hash values across windows to identify redundancies or potential forgeries in the video content. A systematic approach compares hash values for each frame; identical values indicate potential duplicates. To ensure robust detection, a two-level comparison strategy is applied: duplicated frames are identified within and across windows, followed by a detailed frame-to-frame comparison within duplicate groups to verify structural consistency.

**Fig. 6:**
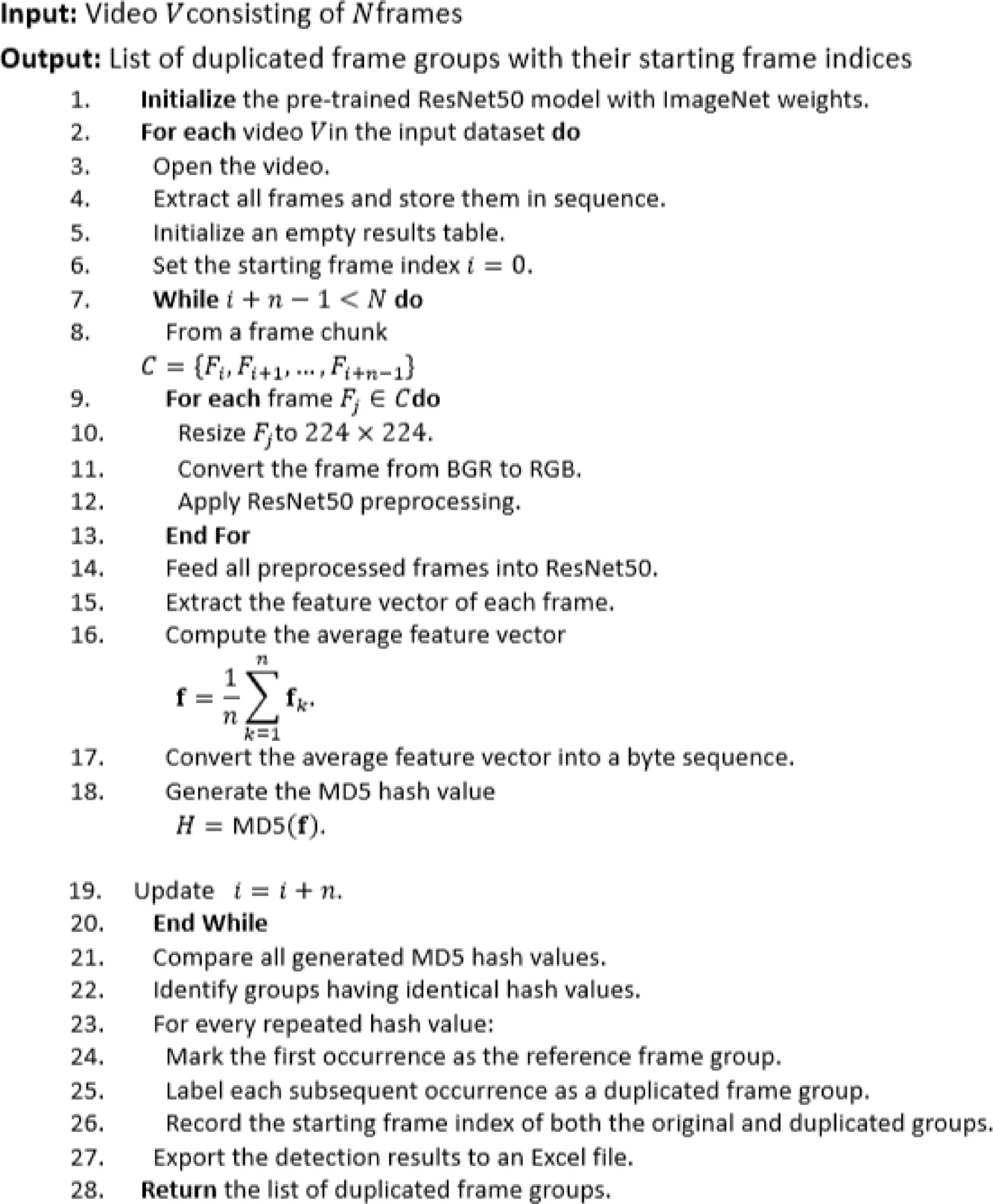
Algorithm of the proposed approach 1 for detecting duplicated frames in video forgery.

The Algorithm in Fig. 6 outlines the proposed method for detecting duplicated frames in videos by extracting feature vectors with ResNet50 and generating unique MD5 hash values for each frame. Frames are processed in fixed-size windows for efficiency, and duplicate frames are identified through hash comparisons both across and within windows, followed by detailed verification. This method offers a scalable and precise solution for detecting structural manipulations in video content.

### Second approach: frame-level feature extraction with temporal validation

**Fig. 7:**
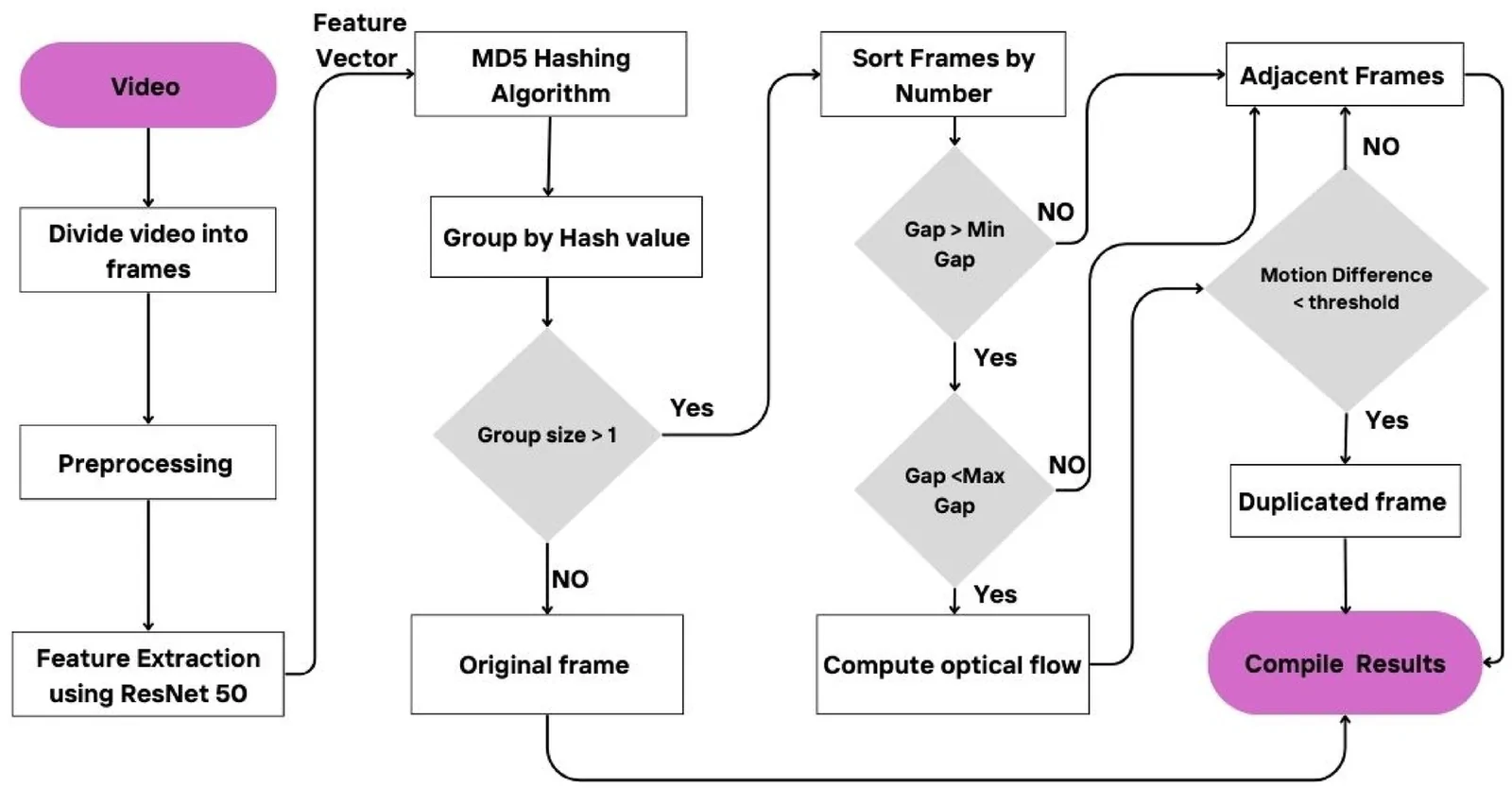
The flowchart of the proposed approach 2 for detecting video duplication forgery.

The proposed method detects duplicated frames in a video by combining deep feature extraction, cryptographic hashing, and temporal validation. A hybrid strategy is employed to enhance forgery detection accuracy through a two-level verification process that integrates hash-based Grouping with temporal validation. The approach consists of five main stages: preprocessing, deep feature extraction using a modified ResNet50 architecture, hash-based candidate identification, temporal filtering, and motion-based validation, as shown in Fig. 7.

#### Modified ResNet50 architecture

This approach extracts features from the conv5_block3_out layer of the ResNet50 architecture. ResNet50 consists of five hierarchical stages, conv1 through conv5, comprising a series of residual units, with the conv5_block3_out layer representing the output of the final unit in the deepest convolutional block. This layer yields a feature tensor of dimensions 7 × 7 × 2048 when given an input of 224 × 224 pixels, providing a rich representation that balances high-level semantics and spatial structure. Unlike the global average pooling layer, the conv5_block3_out layer retains localized activations, making it particularly suitable for detecting subtle tampering artifacts. Spatial average pooling is applied to this tensor to generate a compact yet discriminative 2048-dimensional descriptor for each frame, enabling efficient hashing while preserving critical spatial cues. This choice ensures a balance between computational efficiency and the ability to detect spatially localized manipulations, thereby contributing to the superior performance of the second approach in terms of detection accuracy and false positive reduction^21^.

#### Hash-based grouping

The first level involves a hash-based candidate selection mechanism: each frame is processed with a modified ResNet50 architecture, and the resulting high-dimensional feature vectors are hashed using MD5. A group-by operation on the computed hash values identifies potential duplicates by flagging frames that share the same hash digest.

#### Temporal validation

**Fig. 8:**
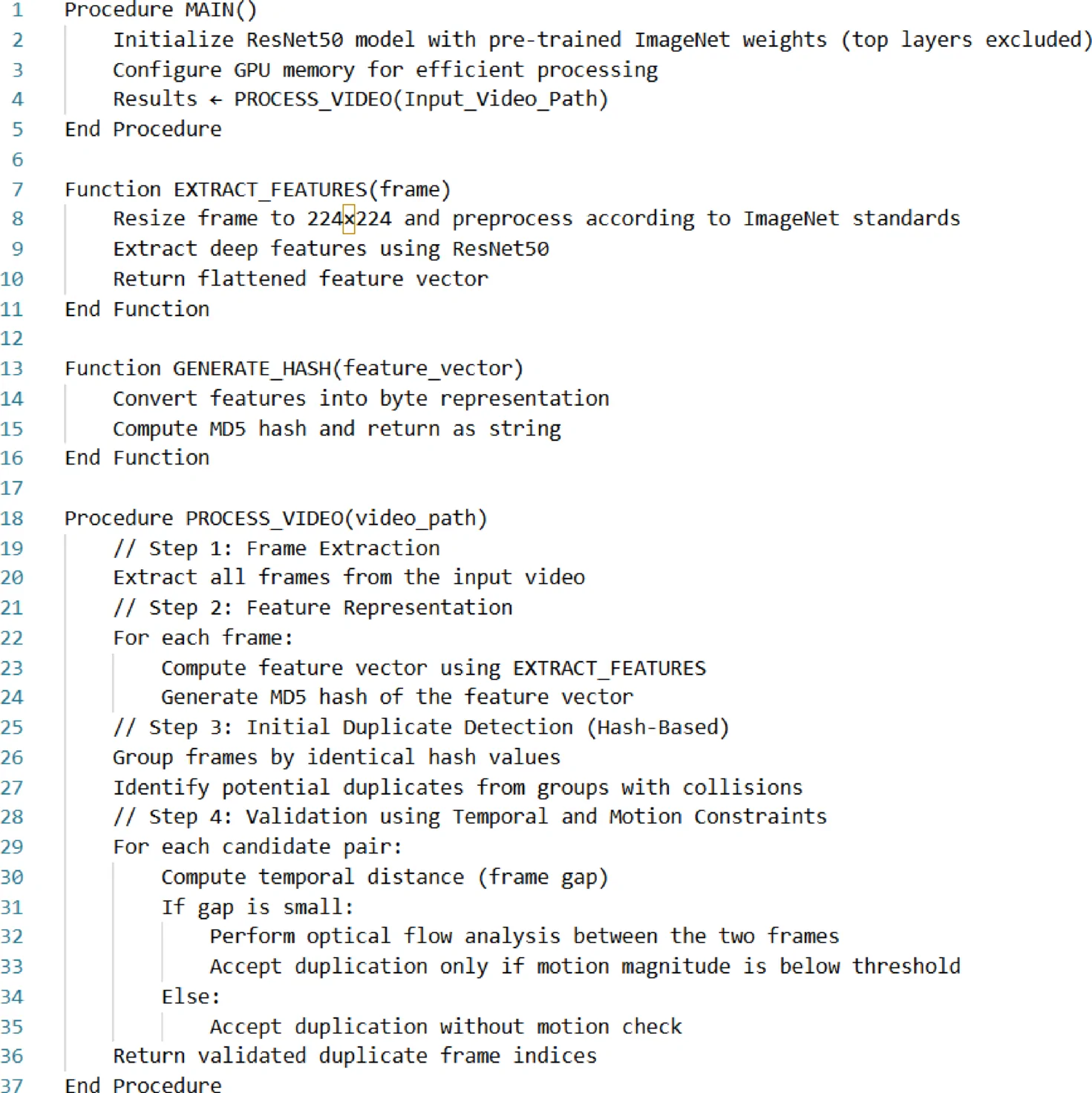
Pseudocode of the proposed approach 2 for detecting duplicated frames in video forgery.

The multi-criteria temporal and structural validation is applied to confirm whether the detected duplicates are indeed forged instances. A gap thresholding mechanism filters out candidate pairs whose frame indices differ by fewer than 30 frames, effectively eliminating natural repetitions such as static scenes or repetitive motion patterns. A spatial similarity check computes cosine similarity between the corresponding feature vectors of candidate pairs, using a variable threshold to retain only structurally consistent duplicates. For candidate pairs within 100 frames of each other, a motion-consistency check is performed using dense optical flow estimation with the Farnebäck algorithm. The magnitude of the computed optical flow field indicates inter-frame motion coherence; a threshold is used to identify pairs exhibiting minimal motion, which are then classified as duplicated content. To optimize computational efficiency, a hybrid strategy is implemented through which all frames are processed individually without window-based segmentation, and optical flow computation is restricted to temporally close candidates^26^. Feature vectors are cached during the initial pass to avoid redundant calculations. The pseudocode in Fig. 8 presents a two-stage validation strategy that includes:

1. *Temporal Gap Filtering*:

For any candidate pair $({F}_{i},{F}_{j})$, if |I–J|< 30, the pair is discarded. This constraint eliminates spurious matches between temporally adjacent frames that may appear visually similar in static scenes.

2. *Cosine Similarity Check*:

The cosine similarity between the feature vectors of frames ${F}_{i} and {F}_{j}$ is calculated to verify candidate duplicates, using the following formula in Eq. 4:

4 $$ Sim\left( {v_{i} ,v_{j} } \right) = \frac{{v_{i} .v_{j} }}{{\left\| {v_{{i{ }}} { }} \right\|.{ }\left\| {v_{{j{ }}} } \right\|}} $$

This metric assesses the angular distance between the two feature vectors, reflecting how semantically similar the frames are. If the similarity score is below a predefined threshold, such as 0.95, the pair is excluded from consideration as a duplicate. This ensures that only frame pairs with highly similar deep representations are retained, thereby reducing false positives resulting from hash collisions that do not accurately reflect true content duplication.

3. *Motion Consistency Validation*:

Motion analysis is applied to frame pairs separated by fewer than 100 frames to confirm the authenticity of candidate duplicates. Dense optical flow is computed between the grayscale representations of the frames using the Farnebäck algorithm^26^, which estimates pixel-wise motion vectors across the image plane. If the computed motion magnitude exceeds a specified threshold, the frame pair is rejected as a duplicate. This validation step effectively filters out scenes with significant motion, ensuring that only visually static or near-identical content is flagged as duplicated, thereby minimizing false positives.

The proposed method’s integration of deep learning and hashing offers transformative societal benefits by addressing critical challenges in video integrity. By achieving high accuracy and faster execution, the framework ensures reliable, scalable detection of manipulated videos, directly combating misinformation in public discourse, legal proceedings, and media ecosystems. Its window-based processing enables real-time analysis of large-scale video data, empowering platforms to swiftly identify forged content such as tampered surveillance footage, thereby safeguarding public trust and security.

Deterministic MD5 hashing minimizes false positives, ensuring accountability in scenarios such as criminal investigations or journalistic verification. Furthermore, the method’s resource-efficient approach democratizes access to forgery detection tools, even for organizations with limited computational infrastructure, fostering equitable protection against digital fraud. These advancements fortify societal resilience against disinformation campaigns, protect individual reputations, and uphold the credibility of digital evidence in an era where video manipulation poses escalating risks to privacy, justice, and democratic processes.

## Experiments and results

The experiments conducted in this study were designed to evaluate the effectiveness and reliability of the proposed approaches for detecting video forgery. They were executed on Google Colab, which provides a powerful Python 3 backend on Google Compute Engine. This setup included access to a GPU with 15.0 GB of dedicated memory, 12.7 GB of system RAM, and 112 GB of hard disk space within a single runtime. The computational resources offered by Google Colab facilitated efficient dataset processing and complex model computations, enabling comprehensive testing of the method’s capabilities. The framework utilizes a pre-trained ResNet50 as a fixed feature extractor, eliminating the need for training-related hyperparameters such as optimizer, learning rate, batch size, and epochs^27^. The experimental design was structured to compare the algorithm’s outputs against ground truth data, analyze its performance using standard metrics, and assess its scalability and applicability to real-world scenarios.

To ensure a fair comparison with recent methods, the proposed framework was evaluated using the same publicly available dataset and experimental protocol as the studies being compared, whenever possible. The comparison focuses primarily on detection performance metrics, including Accuracy, Precision, Recall, and F1-score, rather than execution time.

### Performance parameters

To validate the proposed framework for detecting video forgery, a systematic evaluation was conducted using established ground-truth data. A suite of widely accepted classification metrics was utilized to measure the alignment between the method’s predictions and the actual forgery annotations. These metrics provide comprehensive insight into the algorithm’s ability to detect duplications while minimizing errors.

The primary metrics used included counts of true positives, false positives, true negatives, and false negatives. True positives (TP) represented frames correctly identified as containing forgery, reflecting the method’s sensitivity to duplication. False positives (FPs) are instances in which the technique mistakenly flags original frames as forged, indicating potential over-detection. True negatives (TN) represented frames accurately recognized as original, demonstrating the framework’s ability to disregard unaltered content. Meanwhile, false negatives (FN) were measured in cases where duplicated frames were incorrectly classified as original, indicating missed detections and areas for improvement.

Our experiments are conducted using the following terms for precision, recall, F1-score, and Detection Accuracy:

5 $$ P = \frac{TP}{{TP + FP}} $$

6 $$ R = \frac{TP}{{TP + FN}} $$

7 $$ F1 = 2x\frac{P x R}{{P + R}} $$

8 $$ DA = \frac{TP + FN}{{TP + FP + FN + TN}} $$

Building upon these foundational metrics, additional derived measures were employed to better assess the balance between precision and recall. Precision (P) quantified the framework’s reliability in detecting actual forgeries among all flagged frames, while recall (R) measured its ability to identify all duplications within the dataset. The harmonic meaning of these two, the F1-Score (F1), provides a single-value metric to evaluate the method’s overall effectiveness, particularly in scenarios with imbalanced data. Detection Accuracy (DA) is the proportion of correctly classified frames and provides a broader perspective on the framework’s overall performance.

The evaluation process involved a meticulous frame-by-frame comparison of the proposed method’s predicted outputs with manually annotated ground truth data. For each frame, agreement between the expected and actual labels was classified as either a true positive or a false negative, depending on the frame’s forgery status. Discrepancies contributed to false-positive or false-negative counts, enabling the precise quantification of the framework’s errors. This direct comparison ensured an unbiased assessment of the algorithm’s performance.

In assessing the results, the proposed method demonstrated strong performance across all metrics. It consistently achieved high precision, recall, and F1-score, indicating its ability to detect subtle manipulations while maintaining a low false alarm rate. The method’s accuracy further supported its applicability in real-world scenarios, where false positives and negatives could have significant implications. Comparisons with baseline approaches revealed substantial improvements, particularly in recall, underscoring the proposed method’s enhanced ability to identify duplications.

### Datasets

The Temporal Domain Tampered Video Dataset (TDTVD)^7^ comprises 210 videos, categorized into Event/Object/Person (EOP) tampering and Smart Tampering (ST). The EOP category consists of 120 videos, with 40 per tampering type: frame deletion, frame insertion, and frame duplication. The remaining 90 videos in the ST category involve videos that exhibit various manipulations, including frame deletion, frame insertion, and frame duplication, categorized into three distinct groups: those with 10 frames of forgery, 20 frames of forgery, and 30 frames of forgery, across three different locations within a total of 10 videos for each category. The videos are 320x240 or 640x360, with an average length of 12 seconds, a frame rate of 30 fps, and a bit rate of 3.439 kb/s. The dataset is derived from 16 original videos from SULFA^28^ and 24 from the YouTube VTD dataset^29^, ensuring a diverse range of activities and scenarios for researchers to test their algorithms. Detailed ground truth information is provided for each tampered video, including the type of tampering, the number of affected frames, and their locations, which is crucial for evaluating tampering detection algorithms.

**Fig. 9:**
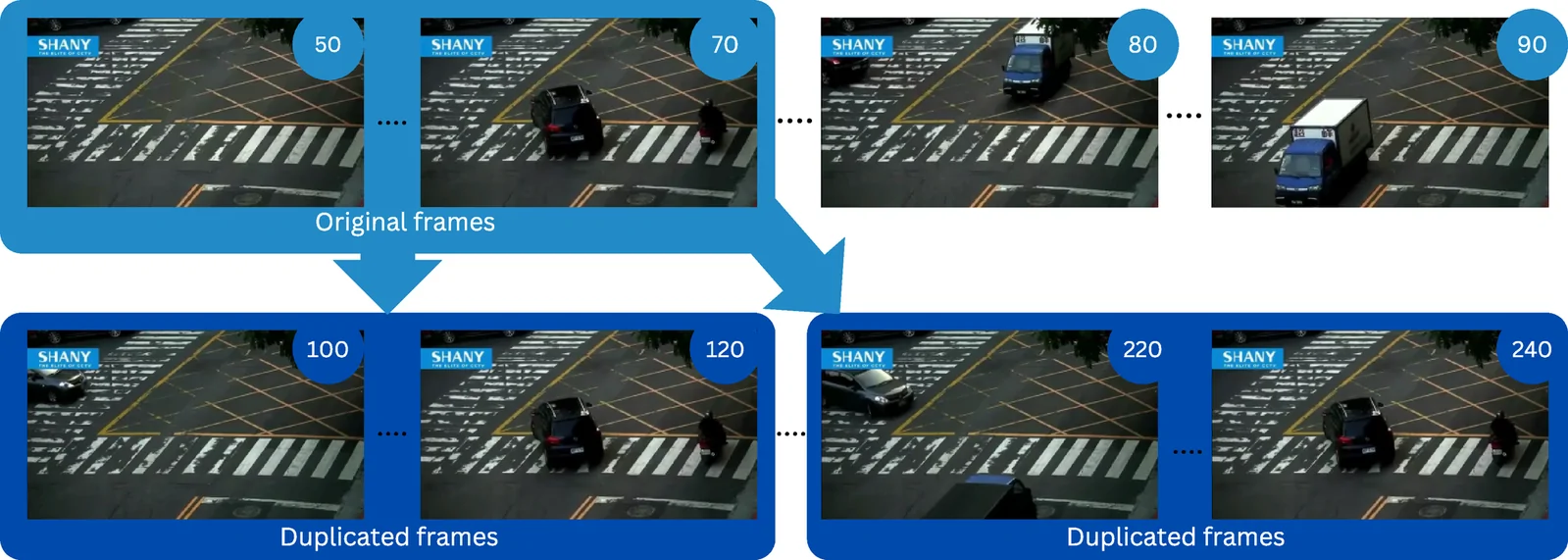
Sample frames of the duplicated forgery video (ST20_10) in the TDTVD dataset.

In addition to outlining the methodologies used for each type of tampering, including frame deletion, duplication, and insertion, all executed using MATLAB. For instance, frame deletion involves selecting specific frames to remove. In contrast, frame duplication involves copying frames and inserting them at different locations to repeat events, as shown in Fig. 9. Frame insertion involves adding frames from another video to maintain continuity and minimize detection. The dataset has been validated against two video tampering detection methods, demonstrating its effectiveness for research on video authentication. The TDTVD dataset is publicly accessible, providing a valuable resource for researchers working on video tampering detection and algorithm development^6^.

Fadl et al.^12^ proposed dataset for inter-frame forgery detection is meticulously curated to evaluate the effectiveness of various detection methods. It includes videos sourced from the SULFA^28^, LASIESTA^30^, and IVY LAB^31^ Datasets, ensuring a diverse mix of resolutions (320 × 240, 352 × 288, 704 × 576) and frame rates (25, 29.97, 30 fps) as shown in Table 1. The dataset includes various types of video forgeries, including frame deletion, insertion, duplication, and shuffling. These manipulations are common in surveillance videos, where the scene and camera position are relatively stable. The dataset comprises 60 test videos, consisting of 28 original and 32 forged, each approximately 10–27 s long. The forged videos have been manipulated to alter the semantic meaning of the original content, providing a realistic challenge for detection algorithms.

**Table 1:** Datasets used for the experiment.

Dataset	Number of videos	Resolution	Average duration	Size of dataset	Bitrate	fps
SULFA	150	320 × 240	10.0 s	6.5 GB	1500 to 2000 Kbps	30
TDTVD	156	320 × 240 or 640 × 360	12.0 s	5.03 GB	3439 kbps	30
Fadl	60	320 × 240 or 352 × 288 or 704 × 576	13.5 s	6.21 GB	55,345 Kbps or 73,026 Kbps	25, 29.9, and 30

**Fig. 10:**
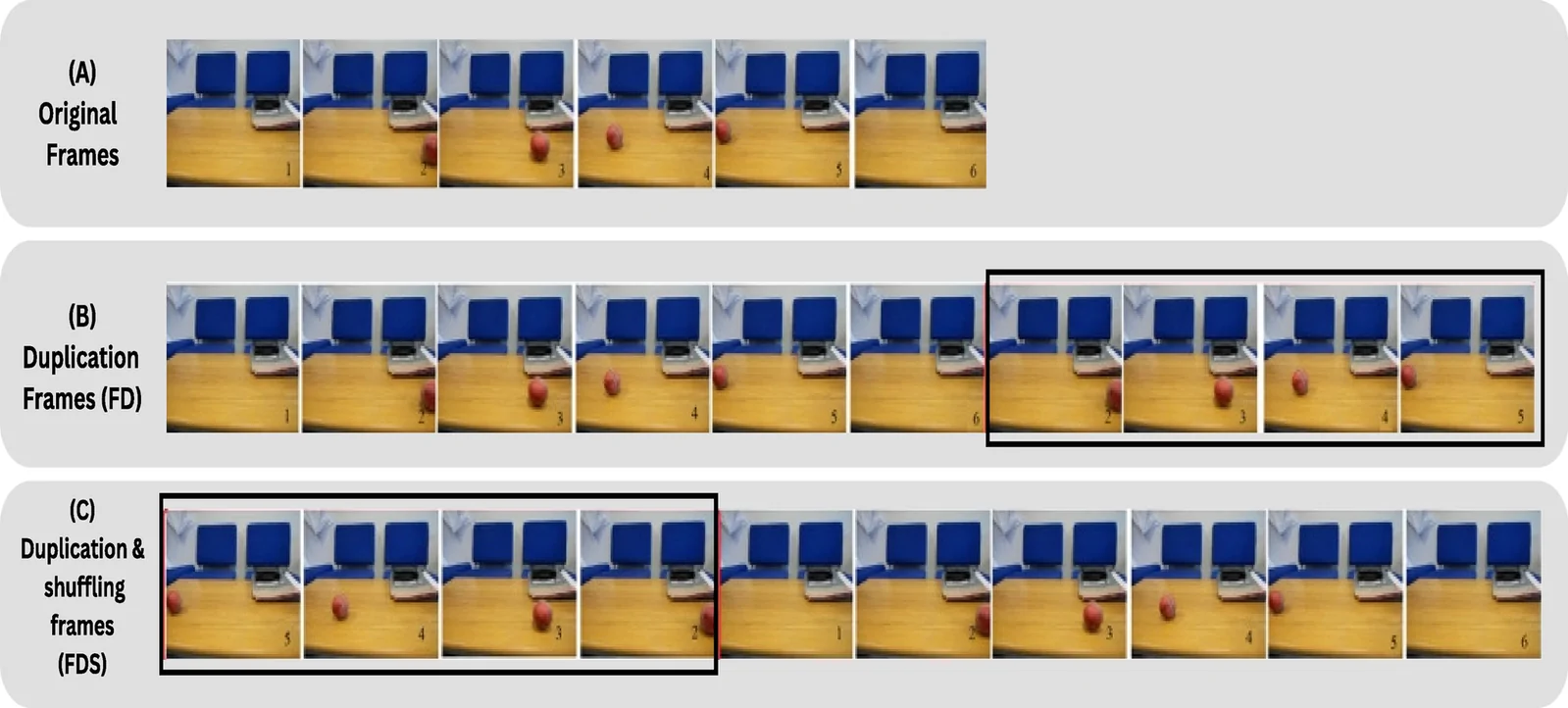
Sample frames from FD and FDS videos in the Fadl dataset.

This dataset is crucial for developing and testing algorithms to detect inter-frame forgeries in surveillance videos. By providing a comprehensive set of scenarios that reflect real-world tampering techniques, it serves as an invaluable resource for researchers in multimedia forensics. Including various types of forgery ensures the dataset can be used to test the robustness and accuracy of different detection methods. These forgery types include frame deletion (FE), where some frames are removed, altering the sequence of events; frame insertion (FI), where foreign frames are added, disrupting the original sequence; frame duplication (FD), where a clip is copied and pasted into another location within the same video; and frame shuffling (FDS), where frames are duplicated and reordered before being pasted, changing the event sequence as shown the difference in Fig. 10. Ultimately, this dataset contributes to the advancement of forensic technology in identifying and mitigating video manipulation, enhancing the reliability and security of video surveillance systems.

### Results

This section presents a comprehensive evaluation of the proposed video duplication detection framework across two benchmark datasets, TDTVD^6^ and Fadl^12^. Results are compared with state-of-the-art hashing and deep learning-based forgery detection methods. A comprehensive sensitivity analysis has been conducted to evaluate the influence of the window size, temporal gap, cosine similarity threshold, and optical flow threshold on the proposed method. Each parameter was varied over a representative range while the remaining parameters were held fixed. Runtime analysis is also conducted to assess the framework’s computational efficiency.

#### TDTVD dataset results

**Fig. 11:**
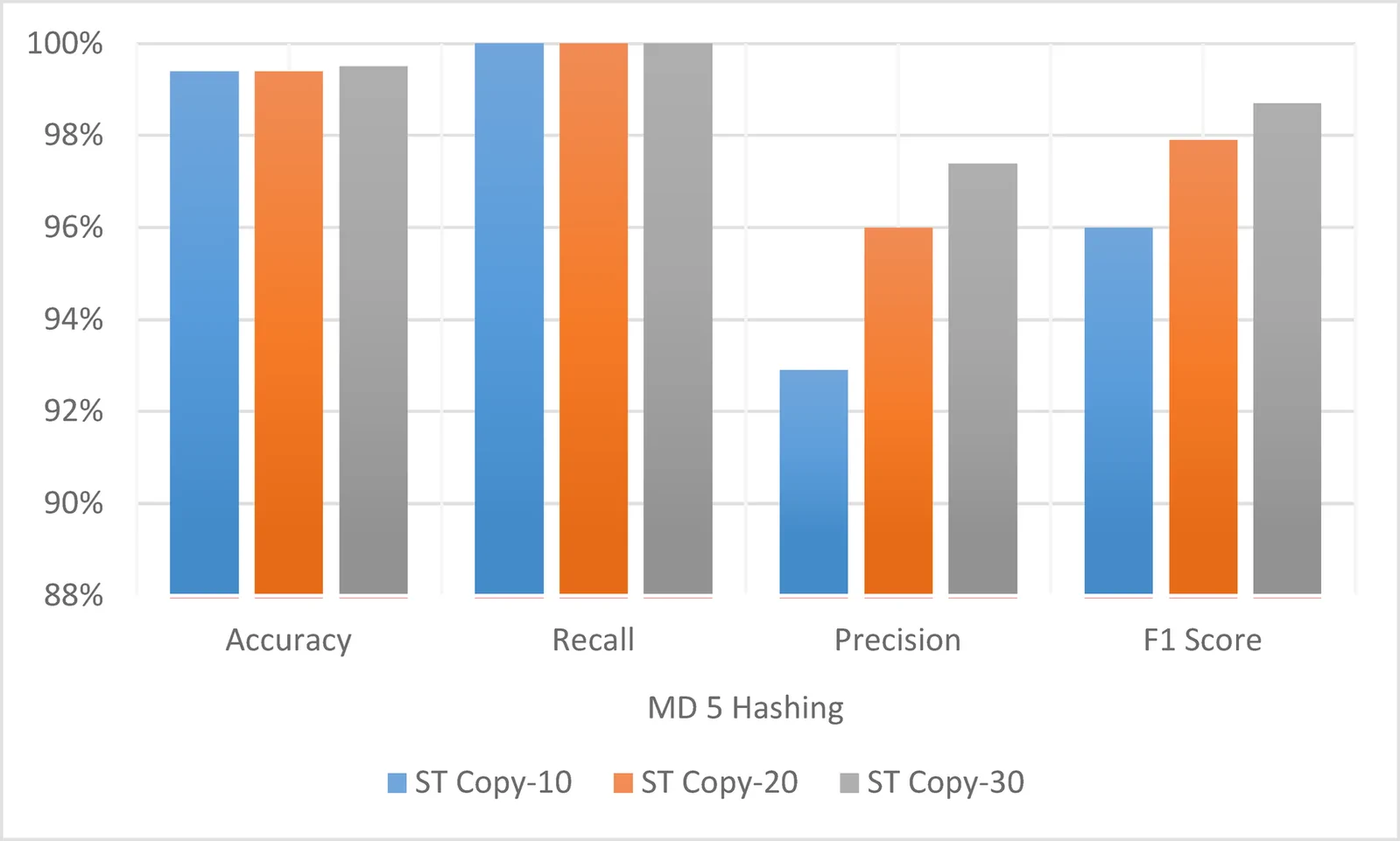
Performance evaluation of the proposed approach 1 for the detection and localization of forged videos on the TDTVD dataset.

**Fig. 12:**
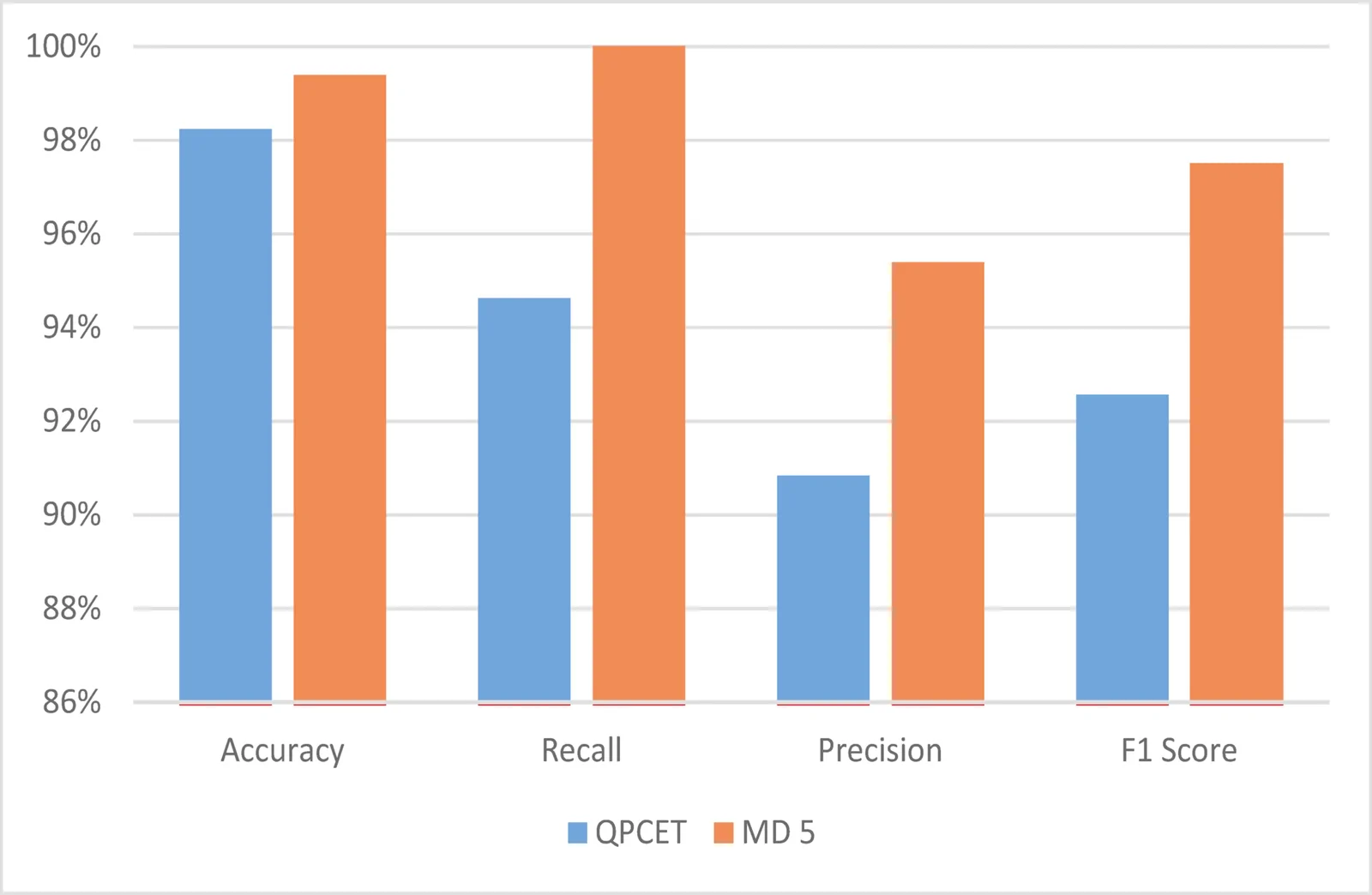
Performance evaluation of the proposed method using the MD5 algorithm against QPCET hashing.

The performance evaluation of the proposed method, which employs the MD5 hashing algorithm, was compared to the QPCET hashing algorithm^32^. Using varying numbers of duplicated frames, as shown in Table 2 and Fig. 11, the experiments were conducted with both the window-based (Approach 1) and group-based (Approach 2) detection pipelines, with results benchmarked against the QPCET hashing method and other techniques. MD5 is highly efficient and rapidly computes unique hashes, making it ideal for real-time analysis and accurately identifying exact, simple frame duplications due to its strict sensitivity to minor data changes^5^. In contrast, QPCET hashing relies on complex mathematical operations that increase computational load, slowing down processing speeds and making it less effective for simple duplication detection^33^. However, what QPCET lacks in speed is made up for by its robustness to complex spatial transformations, such as scaling and rotation, which MD5 cannot handle. MD5 remains the superior choice for fast, straightforward duplication detection, while QPCET is better suited for environments where videos undergo extensive and complex manipulations. In measuring Accuracy and Robustness, the proposed method demonstrated consistently high performance, achieving an average accuracy of 99.4%, surpassing QPCET hashing at 98.2%. The MD5-based approach achieved perfect recall across all experiments, indicating its capability to identify all duplicated frames. QPCET hashing showed a lower average recall of 94.6%. Similarly, the proposed method maintained an average precision of 95.4%, reflecting its reliability in minimizing false positives. The harmonic mean of precision and recall, the F1-score, further confirmed the superiority of the MD5-based method, with an average of 97.5%, compared to 92.6% for QPCET hashing. The MD5 algorithm’s comparative advantage over QPCET hashing across all evaluation metrics is highlighted in Fig. 12.

**Table 2:** Performance evaluation of the proposed method for detecting and localizing forged videos on the TDTVD dataset, compared with QPCET Hashing.

Number of frame duplications	MD5 hashing				QPCET hashing			
	Accuracy	Recall	Precision	F1 score	Accuracy	Recall	Precision	F1 score
ST copy-10	99.40%	100%	92.90%	96.00%	98.10%	90.30%	83.90%	86.70%
ST copy-20	99.40%	100%	96.00%	97.90%	98.30%	96.80%	94.30%	95.50%
ST copy-30	99.50%	100%	97.40%	98.70%	98.30%	96.80%	94.30%	95.50%
Average	99.40%	100%	95.40%	97.50%	98.20%	94.60%	90.80%	92.60%

**Fig. 13:**
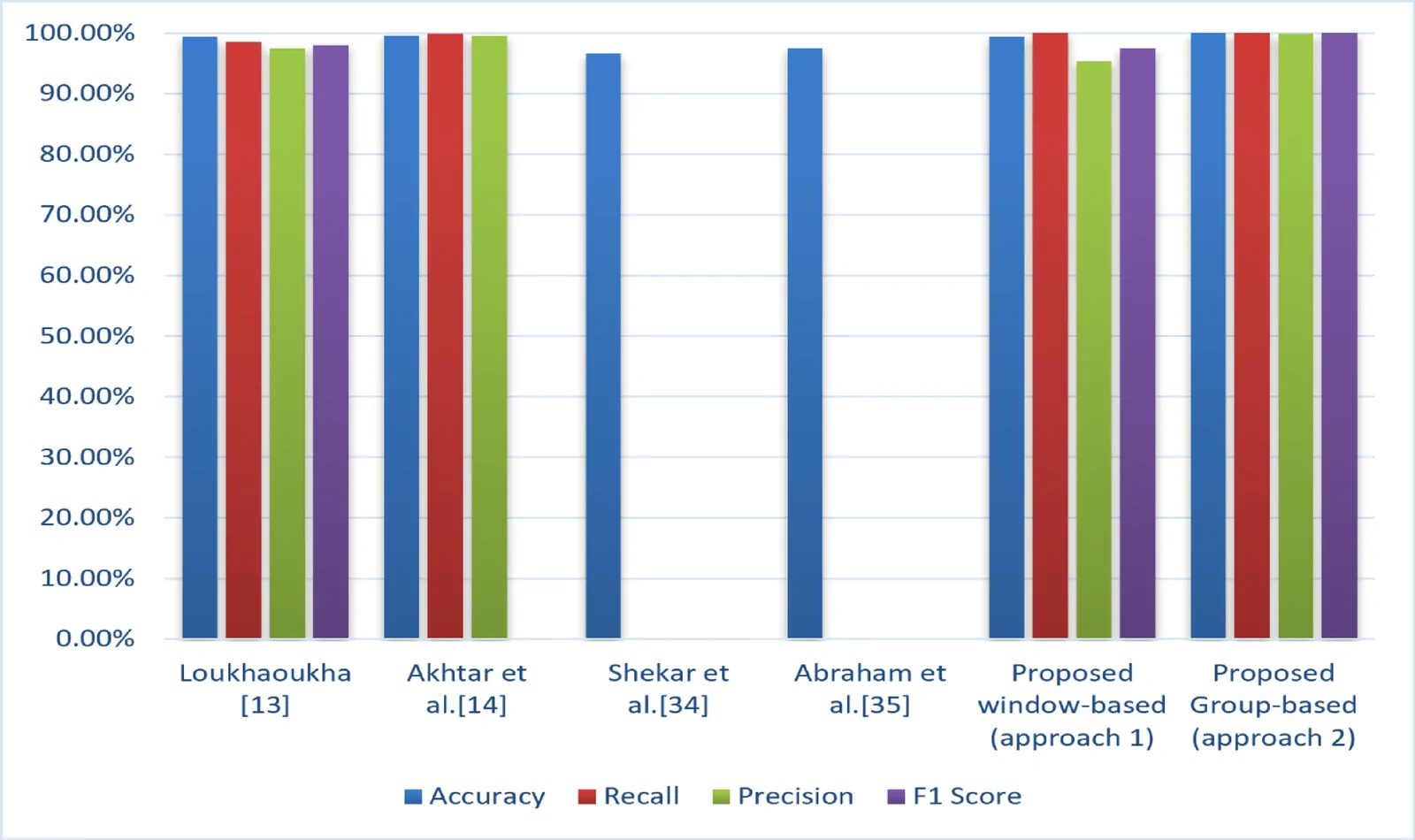
Performance comparison of the proposed approaches with other methods using the TDTVD dataset.

In Table 3, the proposed methods are compared with recent techniques, including those by Loukhaoukha^13^. Akhtar et al.^15^, Shekar et al.^30^, Abraham et al.^31^. The group-based approach (Approach 2) achieves 100% accuracy, recall, and an F1 score of 1.0, indicating ideal detection and classification of duplicated content. The window-based approach also performs competitively, achieving 99.40% accuracy and an F1 score of 97.50%, despite its simpler hash-based design and lack of temporal validation. The overall performance comparison between the proposed approaches and competing methods is illustrated in Fig. 13. These findings confirm that integrating deep feature representations with deterministic hashing yields superior results compared to methods that rely on handcrafted features or classical signal processing techniques.

**Table 3:** Accuracy comparison between the proposed method and other methods on the TDTVD dataset. (Bold refers to the best performance.)

Method	Accuracy	Recall	Precision	F1 score
Loukhaoukha^13^	99.43%	98.58%	97.52%	98.03%
Akhtar et al.^14^	99.57%	99.95%	99.61%	–
Shekar et al.^34^	96.67%	–	–	–
Abraham et al.^35^	97.5%	–	–	–
Proposed window-based (approach 1)	99.40%	**100%**	95.40%	97.50%
Proposed group-based (approach 2)	**100%**	**100%**	**99.91%**	**100%**

The window size parameter was varied, and its effect on accuracy and execution time was recorded to explore the trade-off between detection performance and runtime. As shown in Table 4, smaller window sizes resulted in slightly higher per-frame computation time due to increased batch-processing overhead, yet maintained a stable average accuracy of 99.16%. When the window size exceeded 25 frames, accuracy gradually declined, reaching 98.00% at a window size of 40. This decline is attributed to reduced sensitivity in the hash-matching process, which can obscure local temporal anomalies when using longer windows.

**Table 4:** Performance evaluation of the proposed Approach 1 for varying window size with execution time using the TDTVD dataset. (Bold refers to the selected optimal values.)

Window size	Execution time (S/frame)	Average accuracy
5	0.041	99.16%
10	0.031	99.16%
15	0.031	99.16%
20	0.028	99.16%
**25**	**0.027**	**99.16%**
30	0.026	99.00%
35	0.026	98.42%
40	0.024	98.00%

**Fig. 14:**
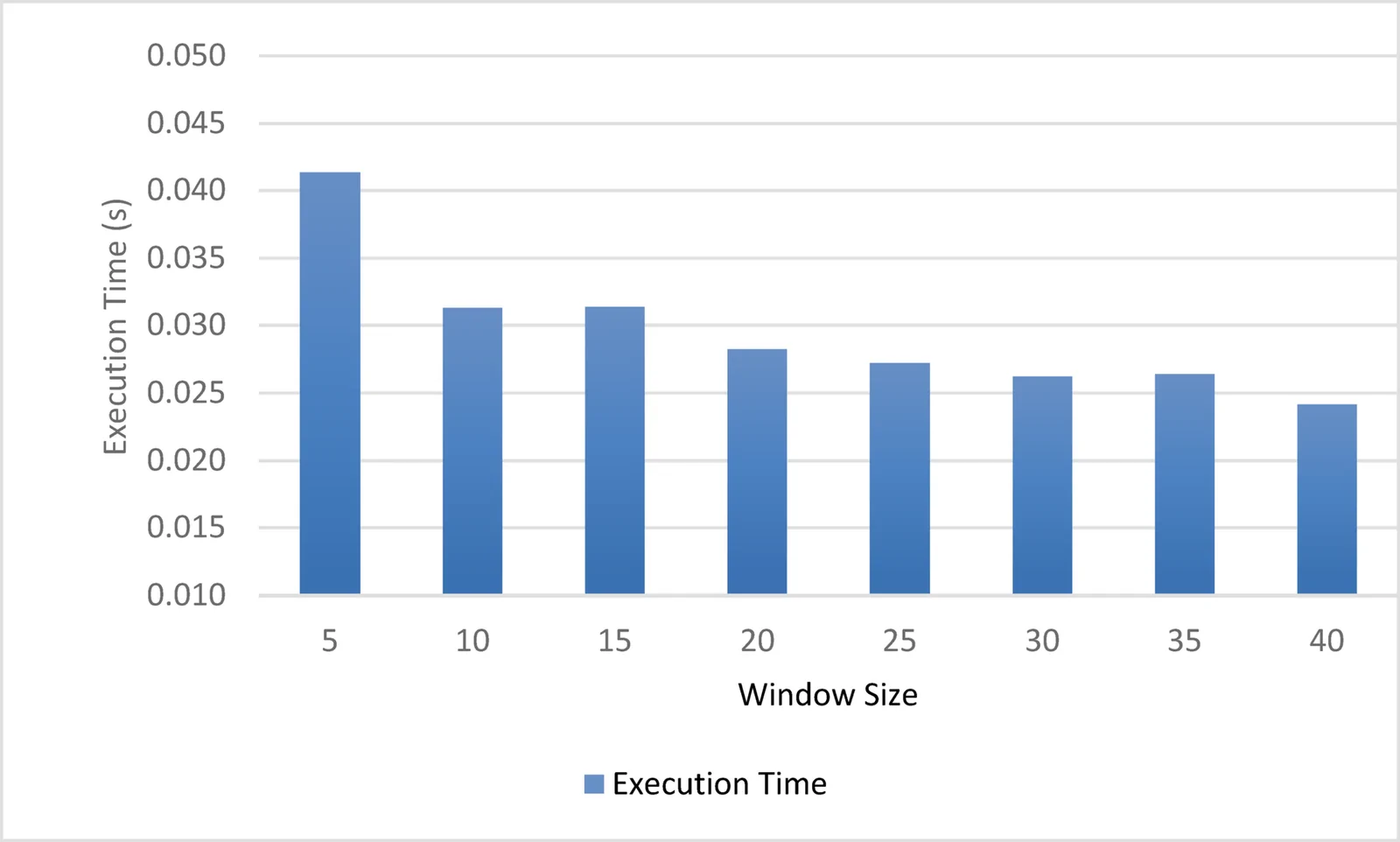
Execution time comparison of the proposed method for varying window size.

**Fig. 15:**
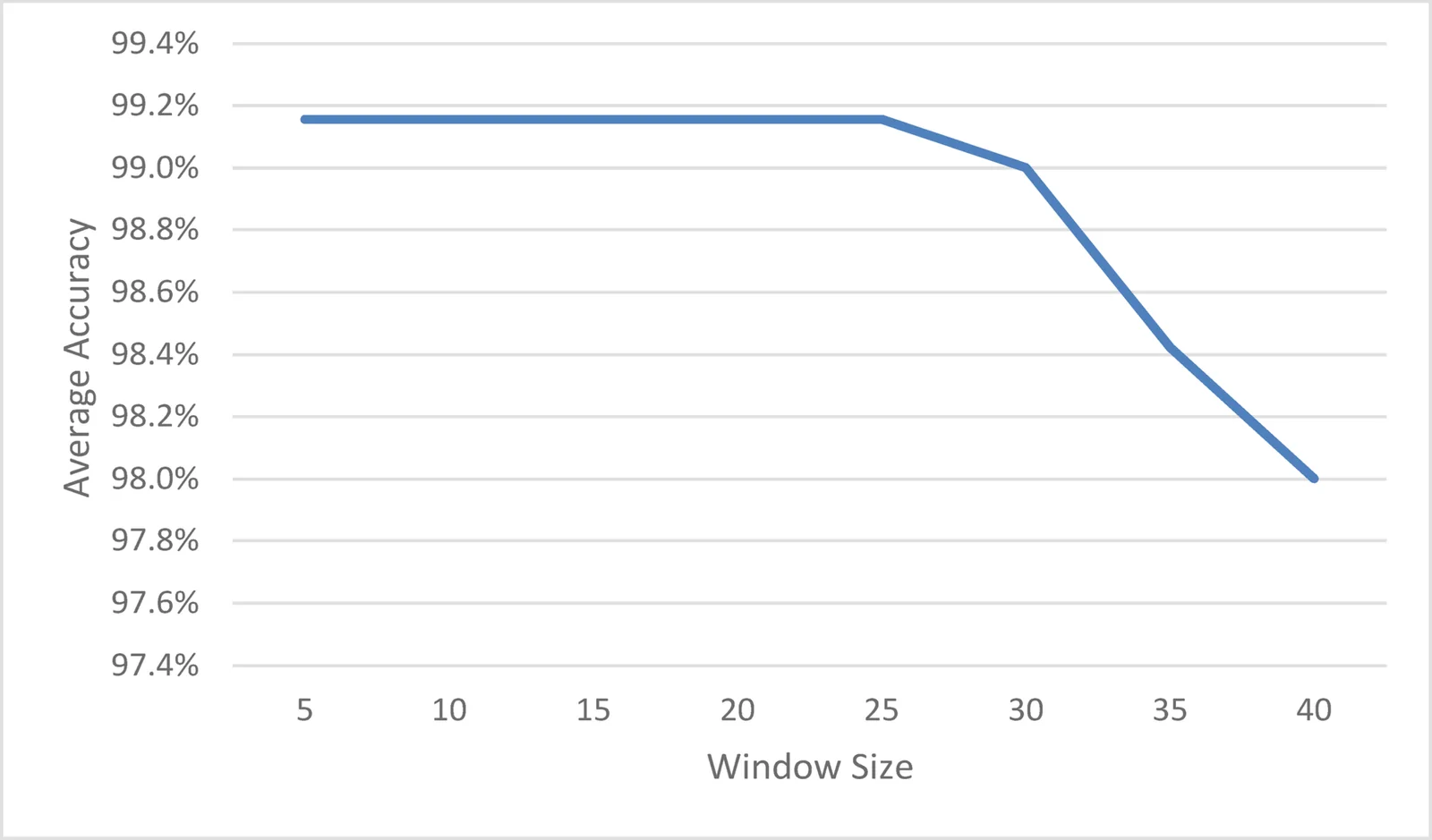
Accuracy comparison of the proposed approach 1 across varying window sizes.

Figures 14 and 15 provide a comparative visualization of accuracy and execution time. The optimal trade-off was observed at a window size of 25 frames, where the method achieved 99.16% accuracy while maintaining an average processing time of 0.027 s per frame. This demonstrates that the proposed window-based approach achieves computational efficiency while maintaining high accuracy, making it highly suitable for real-time applications.

To determine the optimal parameter settings of the proposed framework, a sensitivity analysis was conducted by systematically varying the cosine similarity threshold, the optical flow threshold, and the minimum temporal gap while keeping all other parameters fixed. The objective of this analysis was to identify the parameter values that provide the best tradeoff between detection performance and computational efficiency. As shown in Table 5, all evaluated cosine similarity and optical flow thresholds produced the same detection performance. Therefore, the optimal thresholds were selected based on the shortest execution time. A cosine similarity threshold of 0.95 and an optical flow threshold of 1.0 were adopted, as they achieved the minimum execution times of 0.398 s and 0.380 s, respectively.

**Table 5:** Sensitivity analysis of the cosine similarity and optical flow thresholds in terms of execution time. (Bold refers to the selected optimal values.)

Cosine similarity		Optical flow	
Threshold	Time(S)	Threshold	Time(S)
0.85	0.445	0.5	0.416
0.90	0.433	**1**	**0.380**
**0.95**	**0.398**	1.5	0.395
0.97	0.422	2	0.423

**Fig. 16:**
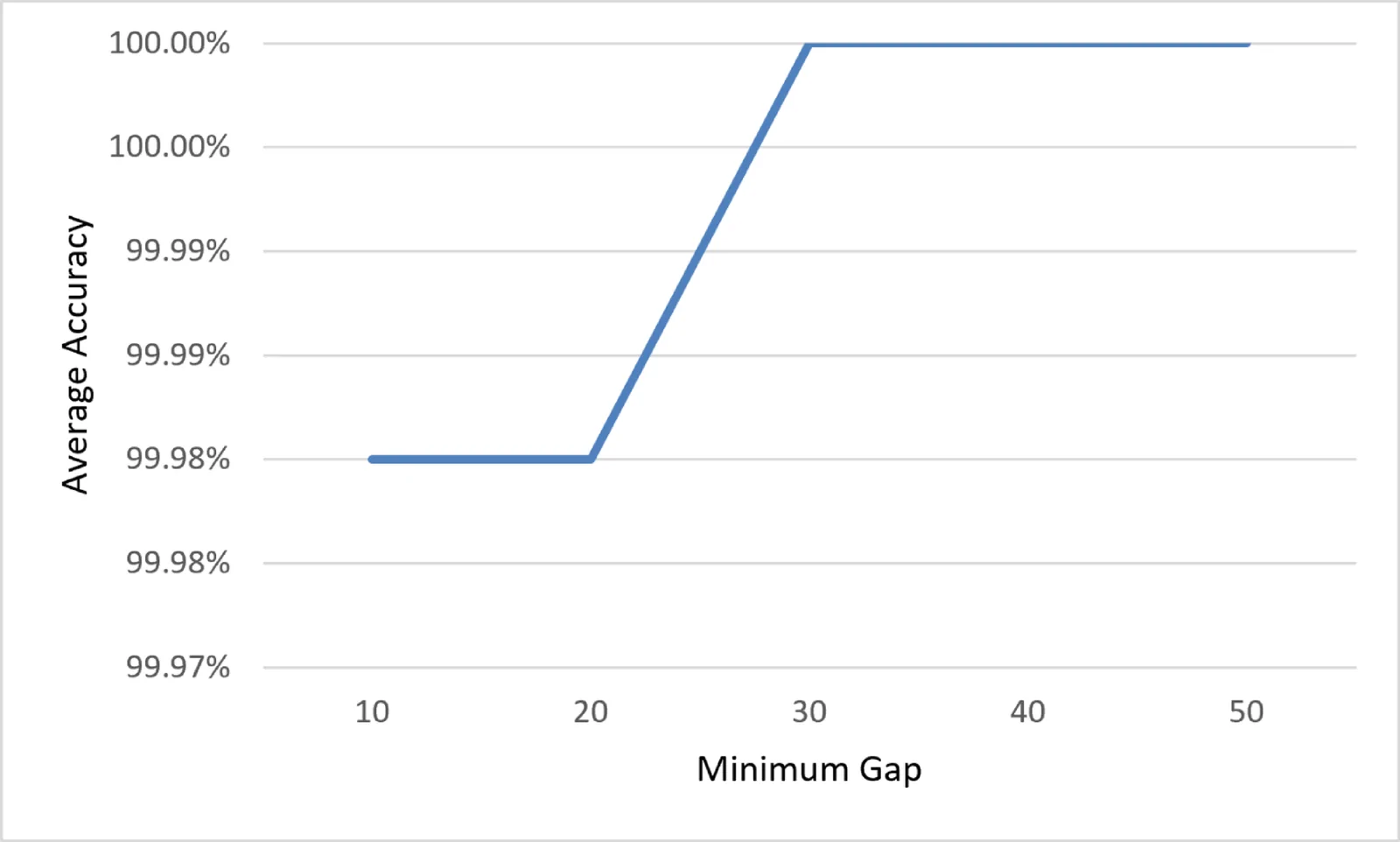
Accuracy comparison of the proposed approach 2 across varying temporal gap sizes.

Table 6 and Fig. 16 present the sensitivity analysis of the minimum temporal gap parameter. The selection criterion was based on computational efficiency. Although temporal gap values of 30, 40, and 50 frames yielded identical minimum execution times, a temporal gap of 30 frames was selected because it is the smallest value that achieves the optimal computational performance while maintaining the highest detection accuracy. This parameter setting was therefore adopted in all subsequent experiments.

**Table 6:** Performance evaluation of the proposed Approach 2 for varying Temporal Gap with execution time using the TDTVD dataset.(Bold refers to the selected optimal values.)

Temporal gap	Execution time (S)	Average accuracy
10	0.437	99.98%
20	0.443	99.98%
**30**	**0.432**	**100.00%**
40	0.394	100.00%
50	0.433	100.00%

#### Fadl and SULFA datasets results

**Fig. 17:**
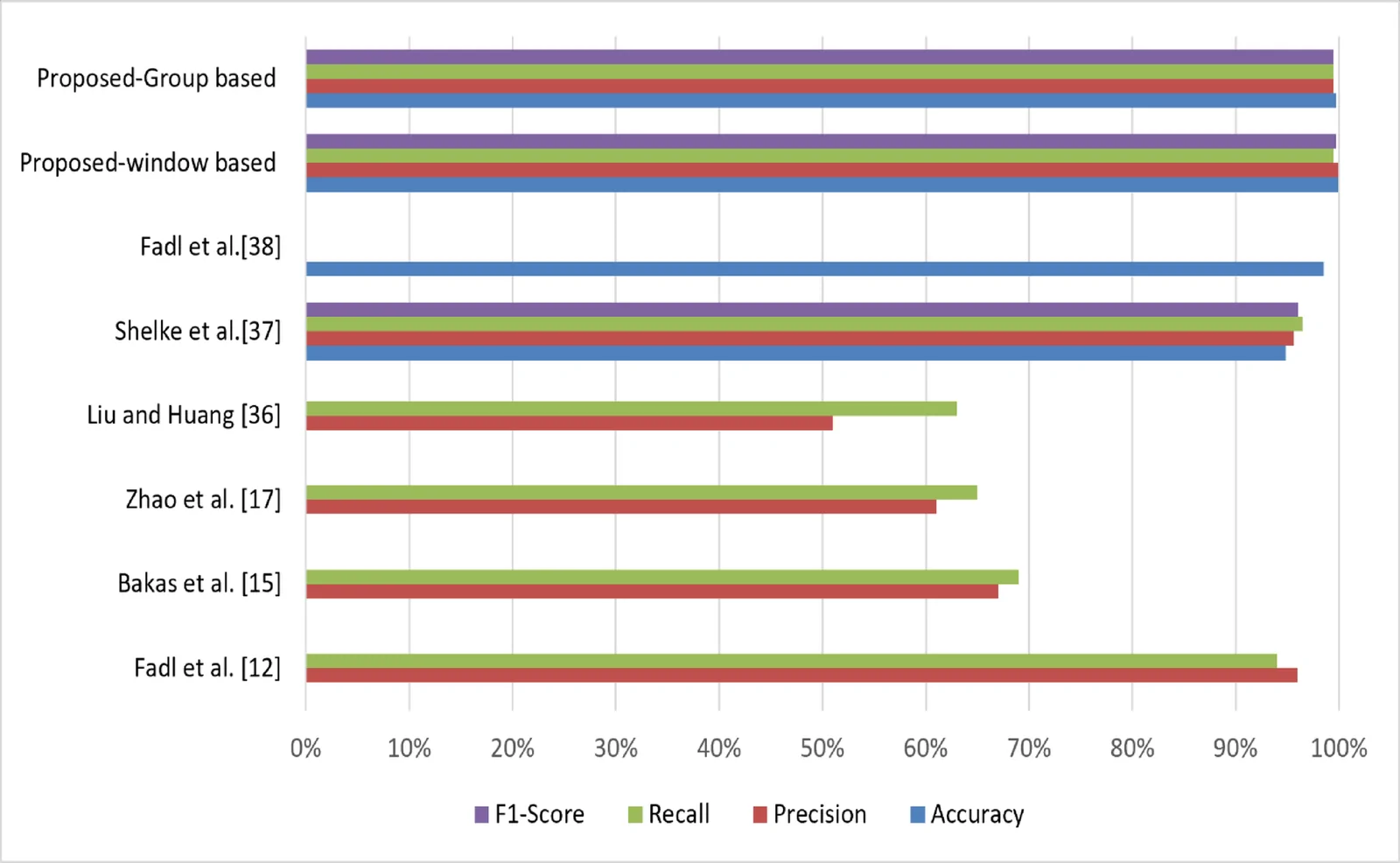
Average performance rates for the proposed method compared with other methods using the Fadl dataset^12^.

The performance of the proposed method was benchmarked against several state-of-the-art techniques using the Fadl dataset^12^ that have been executed on a workstation with an AMD Athlon II X4 641 processor and 8 GB of RAM, as summarized in Fig. 17. The results highlight the method’s superior precision and recall, demonstrating its robustness and adaptability across diverse datasets. The proposed window-based method achieved 99.51% precision and 99.93% recall, demonstrating strong detection performance. In comparison, the group-based method exhibited slightly lower precision while maintaining high recall. This minor drop in precision is attributed to frame-shuffling artifacts in certain videos in the dataset, which introduced structural variations that affected performance. Table 7 represents a substantial improvement over competing approaches, such as those of Zhao et al.^17^ with % 61% precision, % 65% recall, and Bakas et al.^15^ with %67 precision, %69 recall. The results of the proposed window-based method demonstrate its ability to identify duplications while minimizing false positives and negatives accurately.

**Table 7:** Average performance rates for the proposed method compared with other methods using the Fadl dataset^12^. (Bold refers to the best performance)

Method	Accuracy	Precision	Recall	F1-score
Fadl et al.^12^	–	96.00%	94.00%	–
Bakas et al.^15^	–	67.00%	69.00%	–
Zhao et al.^17^	–	61.00%	65.00%	–
Liu and Huang^36^	–	51.00%	63.00%	–
Shelke et al.^37^	94.87%	95.66%	96.48%	96.07%
Fadl et al.^38^	98.50%	–	–	–
Proposed window-based	**99.88%**	**99.93%**	**99.51%**	**99.71%**
Proposed-group based	99.72%	99.48%	99.46%	99.47%

**Fig. 18:**
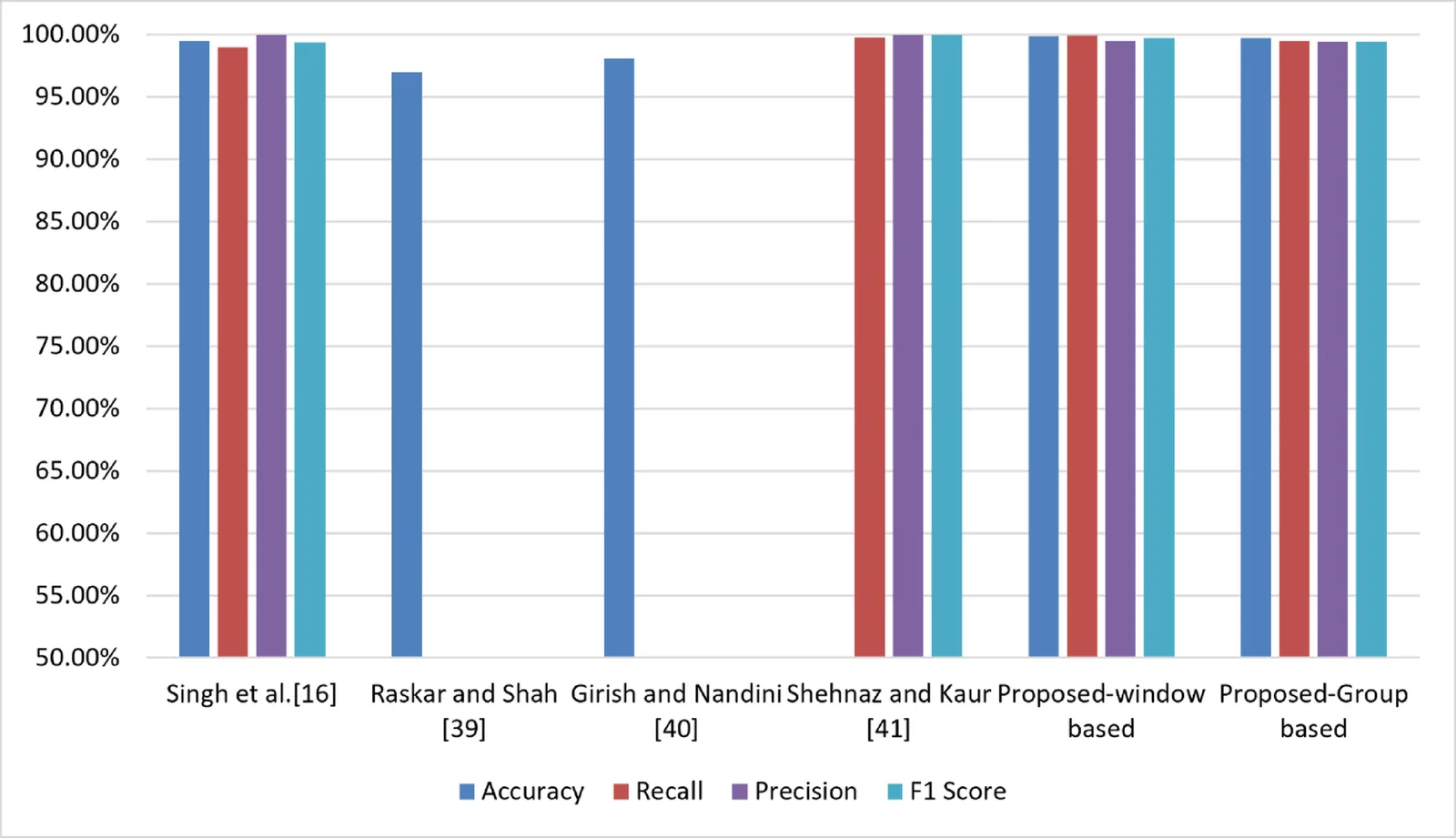
Average performance rates for the proposed method compared with other methods using the SULFA dataset^28^.

These results represent substantial improvements over previous approaches, including Fadl et al.’s method.^12^, which reported 96.00% precision and 94.00% recall, Shelke et al.’s method^33^ with 94.87% accuracy, and Fadl et al.’s alternative implementation^34^, achieving 98.50% accuracy. The consistency of these superior results was further validated on the SULFA dataset^28^, where both proposed implementations maintained their performance advantage, with the window-based approach achieving 99.88% accuracy, 99.93% recall, 99.51% precision, and 99.71% F1-score as shown in Table 8 and illustrated in Fig. 18. This cross-dataset consistency underscores the robustness and generalizability of our framework, particularly when compared to competing methods such as Singh et al.’s approach^16^ which reported 99.5% accuracy, but with less balanced metrics. The minimal performance gap between precision and recall across both implementations indicates our framework’s balanced ability to correctly identify both authentic and tampered video segments, addressing a critical limitation of many existing forensic techniques, which tend to favor one metric over the other.

**Table 8:** Average performance rates for the proposed method compared with other methods using SULFA^28^. (Bold refers to the best performance)

Method	Accuracy	Recall	Precision	F1 score
Singh et al.^16^	99.5%	99.0%	**100%**	99.4%
Raskar and Shah^39^	97.00%	–	–	–
Girish and Nandini^40^	98.13%	–	–	–
Shehnaz and Kaur^41^	–	99.80%	**100%**	**100%**
Proposed-window based	**99.88%**	**99.93%**	99.51%	99.71%
Proposed-group based	99.72%	99.48%	99.46%	99.47%

The proposed method’s consistent, superior metrics underscore its effectiveness in handling varied and complex data conditions. Its ability to generalize across datasets demonstrates its robustness, making it a reliable solution for real-world video forgery detection scenarios. These results establish the proposed method as a significant advancement over existing techniques, reinforcing its applicability and efficiency in diverse applications.

The experimental results confirm that the proposed hybrid framework offers high detection accuracy and scalability across varying video manipulation scenarios. The integration of deep semantic features, deterministic hashing, and selective temporal validation provides a robust solution for frame-level video forgery detection. The group-based approach is effective in minimizing false positives, while the window-based method offers a faster alternative for less sensitive applications.

## Conclusion

The presented approaches offer a scalable, robust solution for detecting duplicated frames in forged videos by combining deep feature extraction and hashing techniques. By leveraging ResNet50 to capture high-dimensional image features and MD5 for deterministic hashing, we enable accurate and efficient identification of frame-level duplications. It achieves 99.4% accuracy on the TDTVD dataset. The integration of window-based processing improves computational efficiency, reducing execution time by 65.85%. This supports real-time analysis of large-scale video datasets and demonstrates potential for practical applications in forgery detection across diverse security and integrity verification scenarios, with performance metrics including precision, recall, F1 Score, and detection accuracy. The framework’s robustness is validated on the SULFA and Fadl datasets, demonstrating flexibility across various forgery scenarios with 99% precision and recall. Despite its strengths, the proposed method faces challenges in detecting duplicated frames in forged videos. Although the proposed framework was validated independently on three publicly available datasets, a comprehensive assessment using unseen datasets would provide stronger evidence of its generalization capability. The use of a pre-trained ResNet50 feature extractor was motivated by the limited availability of large-scale annotated datasets for frame duplication detection, thereby avoiding the need for dataset-specific model training. Additionally, real-time video content manipulations pose significant challenges, particularly when reference videos are unavailable. Moreover, processing large volumes of video data demands substantial computational resources.

Future research could expand the method’s applicability to emerging challenges. Integrating unsupervised learning or contrastive learning could enhance detection in scenarios such as real-time social media monitoring. Exploring hybrid architectures that combine attention mechanisms with temporal analysis could further address complex manipulations, such as adversarial attacks or AI-generated videos. Expanding training datasets to include synthetic forgeries from GANs or diffusion models would enhance generalization, ensuring resilience against evolving tampering techniques. These advancements would reinforce the method’s role as a foundational tool for combating misinformation and securing digital media integrity in dynamic threat landscapes.

## Data Availability

The datasets analyzed during the current study are as follows: (a) SULFA—available at https://sites.google.com/site/rewindpolimi/downloads/datasets/video-copy-move-forgeriesdataset (b) The TDTVD dataset (Forged/Tampered video dataset) developed and analyzed during the current study is available at https://doi.org/10.1007/s11042-020-09205-w. (c) Fadl Dataset available at https://drive.google.com/open?id=1ryMNJvKDaa7y187O1Y1CEjr4FxTSsVq9.

## References

[1] Diwan, A., Dixit, S., Subbiah, R. & Mahadeva, R. Systematic analysis of video tampering and detection techniques. *Cogent Eng.* **11**, 2424466 (2024).

[2] Ding, B., Fan, Z., Zhao, Z. & Xia, S. Mining collaborative spatio-temporal clues for face forgery detection. *Multim. Tools Appl.* **83**, 27901–27920. 10.1007/s11042-023-16173-4 (2024).

[3] Ali, M. M. et al. Interframe forgery video detection: Datasets, methods, challenges, and search directions. *Electronics* **14**, 2680 (2025).

[4] Ulutas, G., Ustubioglu, B., Ulutas, M. & Nabiyev, V. V. Frame duplication detection based on bow model. *Multim. Syst.* **24**, 549–567 (2018).

[5] Ren, H., Atwa, W., Zhang, H., Muhammad, S. & Emam, M. Frame duplication forgery detection and localization algorithm based on the improved Levenshtein distance. *Sci. Program.* 10.1155/2021/5595850 (2021).

[6] Panchal, H. D. & Shah, H. B. Video tampering dataset development in temporal domain for video forgery authentication. *Multim. Tools Appl.* **79**, 24553–24577 (2020).

[7] Patel, J. & Sheth, R. An optimized convolution neural network based inter-frame forgery detection model-a multi-feature extraction framework. *ICTACT J. Image Video Process.* **12**, 2570–2581 (2021).

[8] Soomro, Zamir, A. R., Shah, M. UCF101: A dataset of 101 human actions classes from videos in the wild. Available online: https://www.crcv.ucf.edu/data/UCF101.php (Accessed on 7-2025).

[9] Munawar, M. & Noreen, I. Duplicate frame video forgery detection using Siamese-based RNN. *Intell. Autom. Soft Comput.* **29**, 927–937. 10.32604/iasc.2021.018854 (2021).

[10] Kumar, V., Kansal, V. & Gaur, M. Multiple forgery detection in video using convolution neural network. *Comput. Mater. Contin.* 10.32604/cmc.2022.023545 (2022).

[11] Nguyen, X. H., Hu, Y. VIFFD-A dataset for detecting video inter-frame forgeries. Available online: https://data.mendeley.com/datasets/r3ss3v53sj/6 (Accessed on 7–2025).

[12] Fadl, S., Han, Q. & Qiong, L. Exposing video inter-frame forgery via histogram of oriented gradients and motion energy image. *Multidimens. Syst. Signal Process.* **31**, 1365–1384 (2020).

[13] Loukhaoukha, K. Frame duplication forgery detection and localization based on QR decomposition and Minkowski distance. *J. Forensic Sci.* 10.1111/1556-4029.70043 (2025). 40357982

[14] Akhtar, N., Hussain, M. & Habib, Z. Two–stage detection and localization of inter–frame tampering in surveillance videos using texture and optical flow. *Mathematics* **12**, 3482 (2024).

[15] Bakas, J., Naskar, R. & Dixit, R. Detection and localization of inter-frame video forgeries based on inconsistency in correlation distribution between Haralick coded frames. *Multim. Tools Appl.* **78**, 4905–4935 (2019).

[16] Singh, G. & Singh, K. Video frame and region duplication forgery detection based on correlation coefficient and coefficient of variation. *Multim. Tools Appl.* **78**, 11527–11562. 10.1007/s11042-018-6585-1 (2019).

[17] Zhao, D.-N., Wang, R.-K. & Lu, Z.-M. Inter-frame passive-blind forgery detection for video shot based on similarity analysis. *Multim. Tools Appl.* **77**, 25389–25408 (2018).

[18] Sadeghi-Nasab, A. & Rafe, V. A comprehensive review of the security flaws of hashing algorithms. *J. Comput. Virol. Hacking Tech.* **19**, 287–302 (2023).

[19] Vadivelu, A. A., Maithili Jha, M.L., Maitri S. Image forgery detection using OpenCV and MD5. *J. Emerg. Technol. Innov. Res.* **10** (2023).

[20] Lian, N., Li, J., Wang, J., Luo, R., Wang, Y., Xia, S.-T., Chen, B. AutoSSVH: Exploring automated frame sampling for efficient self-supervised video hashing. In *Proceedings of the proceedings of the computer vision and pattern recognition conference*, 18881–18890 (2025).

[21] Ahsan, M. M. et al. Enhancing monkeypox diagnosis and explanation through modified transfer learning, vision transformers, and federated learning. *Inform. Med. Unlocked* **45**, 101449 (2024).

[22] Singh, U., Rathor, S., Kumar, M. Deep video forensics: unveiling spatial forgery detection with modified ResNet50. In *Proceedings of the 2024 IEEE 13th international conference on communication systems and network technologies (CSNT)*, 260–265 (2024).

[23] Al-Banhawy, N. H., Mohsen, H. & Ghali, N. I. Signature identification and verification systems: A comparative study on the online and offline techniques. *Future Comput. Inform. J.* **5**(1), 28–45. 10.54623/fue.fcij.5.1.3 (2020).

[24] Qazi, E. U. H., Zia, T. & Almorjan, A. Deep learning-based digital image forgery detection system. *Appl. Sci.* **12**, 2851 (2022).

[25] He, K., Zhang, X., Ren, S., Sun, J. Deep residual learning for image recognition. In *Proceedings of the proceedings of the IEEE conference on computer vision and pattern recognition*, 770–778 (2016).

[26] Farnebäck, G. Two-frame motion estimation based on polynomial expansion. In Proceedings of the Scandinavian conference on Image analysis, 2003; pp. 363–370.

[27] Keras ResNet50. Available online: https://github.com/keras-team/keras/blob/v3.3.3/keras/src/applications/resnet.py#L382-L418 (Accessed on 7–2026).

[28] Sulfa dataset. Available online: http://sulfa.cs.surrey.ac.uk/forged.php (Accessed on 2020–07–08).

[29] Al-Sanjary, O. I., Ahmed, A. A. & Sulong, G. Development of a video tampering dataset for forensic investigation. *Forensic Sci. Int.* **266**, 565–572 (2016). 27574113 10.1016/j.forsciint.2016.07.013

[30] Cuevas, C., Yáñez, E. M. & García, N. Labeled dataset for integral evaluation of moving object detection algorithms: LASIESTA. *Comput. Vis. Image Underst.* **152**, 103–117 (2016).

[31] Sohn, H., De Neve, W. & Ro, Y. M. Privacy protection in video surveillance systems: Analysis of subband-adaptive scrambling in JPEG XR. *IEEE Trans. Circuits Syst. Video Technol.* **21**, 170–177 (2011).

[32] Hosny, K. M., Khedr, Y. M., Khedr, W. I. & Mohamed, E. R. Robust color image hashing using quaternion polar complex exponential transform for image authentication. *Circuits Syst. Signal Process.* **37**, 5441–5462. 10.1007/s00034-018-0822-8 (2018).

[33] Li, L., Lu, J., Zhang, S., Mohaisen, L. & Emam, M. Frame duplication forgery detection in surveillance video sequences using textural features. *Electronics* **12**, 4597 (2023).

[34] Shekar, B., Abraham, W., Pilar, B. A simple difference based inter frame video forgery detection and localization. In *Proceedings of the international conference on soft computing and its engineering applications*, pp. 3–15 (2023).

[35] Abraham, W., Shekar, B.H., Pilar, B. Inter-frame video forgery detection—A PCA-based approach. In *Proceedings of the fifth international conference on computing and network communications*, Singapore, 365–377 (2025).

[36] Li, H., Luo, W., Qiu, X. & Huang, J. Image forgery localization via integrating tampering possibility maps. *IEEE Trans. Inf. Forensics Secur.* **12**, 1240–1252 (2017).

[37] Shelke, N. A. & Kasana, S. S. Multiple forgeries identification in digital video based on correlation consistency between entropy coded frames. *Multim. Syst.* **28**, 267–280. 10.1007/s00530-021-00837-y (2022).

[38] Fadl, S., Han, Q. & Li, Q. CNN spatiotemporal features and fusion for surveillance video forgery detection. *Signal Process. Image Commun.* **90**, 116066. 10.1016/j.image.2020.116066 (2021).

[39] Raskar, P. S. & Shah, S. K. VFDHSOG: Copy-move video forgery detection using histogram of second order gradients. *Wirel. Pers. Commun.* **122**, 1617–1654. 10.1007/s11277-021-08964-5 (2022).

[40] Girish, N. & Nandini, C. Inter-frame video forgery detection using UFS-MSRC algorithm and LSTM network. *Int. J. Model. Simul. Sci. Comput.* **14**, 2341013. 10.1142/s1793962323410131 (2023).

[41] Shehnaz, Kaur, M. Detection and localization of multiple inter-frame forgeries in digital videos. *Multimedia Tools Appl.*, **83**, 71973–72005, 10.1007/s11042-024-18263-3 (2024).

